# *Arabidopsis* seed-specific vacuolar aquaporins are involved in maintaining seed longevity under the control of *ABSCISIC ACID INSENSITIVE 3*

**DOI:** 10.1093/jxb/erv244

**Published:** 2015-05-26

**Authors:** Zhilei Mao, Weining Sun

**Affiliations:** Institute of Plant Physiology and Ecology, Shanghai Institutes for Biological Sciences, Chinese Academy of Sciences, Fenglin Road 300, Shanghai, 200032, People’s Republic of China

**Keywords:** ABI3, *Arabidopsis*, hydrogen peroxide, seed longevity, TIP3.

## Abstract

The tonoplast intrinsic proteins TIP3;1 and TIP3;2 are specifically expressed during seed maturation and localized to the seed protein storage vacuole membrane. However, the function and physiological roles of TIP3s are still largely unknown. The seed performance of *TIP3* knockdown mutants was analysed using the controlled deterioration test. The *tip3;1/tip3;2* double mutant was affected in seed longevity and accumulated high levels of hydrogen peroxide compared with the wild type, suggesting that TIP3s function in seed longevity. The transcription factor ABSCISIC ACID INSENSITIVE 3 (ABI3) is known to be involved in seed desiccation tolerance and seed longevity. *TIP3* transcript and protein levels were significantly reduced in *abi3-6* mutant seeds. *TIP3;1* and *TIP3;2* promoters could be activated by ABI3 in the presence of abscisic acid (ABA) in *Arabidopsis* protoplasts. TIP3 proteins were detected in the protoplasts transiently expressing *ABI3* and in *ABI3*-overexpressing seedlings when treated with ABA. Furthermore, ABI3 directly binds to the RY motif of the *TIP3* promoters. Therefore, seed-specific TIP3s may help maintain seed longevity under the expressional control of ABI3 during seed maturation and are members of the ABI3-mediated seed longevity pathway together with small heat shock proteins and late embryo abundant proteins.

## Highlight

*Arabidopsis* tonoplast intrinsic protein TIP3;1 and TIP3;2 are shown to play a role in seed longevity under the transcriptional control of ABI3.

## Introduction

Tonoplast intrinsic proteins (TIPs), which belong to the major intrinsic protein family, are members of plant aquaporins (AQPs) and are localized in the membrane of the vacuole. These proteins play critical roles in the transport of water and small neutral substrates such as glycerol, urea, ammonia, and hydrogen peroxide (H_2_O_2_) (Liu *et al.*, 2003; Loque *et al.*, 2005; Bienert *et al.*, 2007; Dynowski *et al.*, 2008). The TIP family consists of five subgroups, namely TIP1 (γ-TIP), TIP2 (δ-TIP), TIP3 (α-TIP and β-TIP), TIP4 (ε-TIP), and TIP5 (ξ-TIP). TIP isoforms show different temporal and spatial expression patterns. TIP1s (TIP1;1 and TIP1;2) are vegetative TIPs localized to lytic vacuoles, whereas TIP3s are seed-specific TIPs localized to seed protein storage vacuoles (PSVs) (Hofte *et al.*, 1992; Ludevid *et al.*, 1992; Gattolin *et al.*, 2011). *AtTIP1;3* and *AtTIP5;1* are thought to be specifically expressed in pollen and localized to the vegetative vacuole and sperm vacuole, respectively (Wudick *et al.*, 2014).

α-TIP was first purified and identified from *Phaseolus vulgaris* cotyledons, and the water channel activity of α-TIP is regulated by its phosphorylation (Johnson *et al.*, 1989; Maurel *et al.*, 1995). This protein is specifically expressed during seed maturation and is most abundantly accumulated in dry mature seeds. Immunoelectron microscopy experiments revealed its specific localization to the membranes of seed PSVs in cotyledons and axes (Johnson *et al.*, 1989). α-TIPs are highly conserved proteins that are widely distributed in dicots and monocots (Chaumont *et al.*, 2001; Sakurai *et al.*, 2005; Reuscher *et al.*, 2013; Zhang *et al.*, 2013). *Arabidopsis* contains two α-TIP orthologues, *TIP3;1* (also known as α-TIP) and *TIP3;2* (also known as β-TIP). Recently, using fluorescent protein-fused TIP3s, Gattolin *et al*. demonstrated that TIP3s are dual localized to both the tonoplast and plasma membrane during seed maturation and seed germination (Gattolin *et al.*, 2011).

Single *TIP* gene loss-of-function mutants do not show obvious phenotypes, probably due to the functional redundancy between different TIPs. The *tip1;3/tip5;1* double knockout mutant displays an abnormal rate of barren siliques, indicating that TIPs expressed specifically in pollen contribute to plant reproduction (Wudick *et al.*, 2014). TIP3;1 and TIP3;2 are seed-specific TIP3 isoforms in *Arabidopsis* and may be the only types of AQPs in mature embryos (Gattolin *et al.*, 2011). Screening of the directly regulated targets of ABSCISIC ACID INSENSITIVE 3 (ABI3), which is a master regulator that controls seed maturation (Parcy *et al.*, 1994; Suzuki and McCarty, 2008), by genome-wide chromatin immunoprecipitation (ChIP-chip) revealed 98 *Arabidopsis* genes including *TIP3;1* and *TIP3;2* as targets (Monke *et al.*, 2012). The transcription factor ABI3 protein contains a plant-specific DNA-binding domain, designated as the B3 domain (McCarty *et al.*, 1991; Giraudat *et al.*, 1992), and activates numerous seed-specific genes. *abi3* mutants exhibit pleiotropic phenotypes to various degrees, depending on the *ABI3* alleles, such as desiccation intolerance, decreased seed longevity, abscisic acid (ABA) insensitivity, and lack of chlorophyll degradation (Koornneef *et al.*, 1984; Ooms *et al.*, 1993; Rohde *et al.*, 2000; Tesnier *et al.*, 2002; Delmas *et al.*, 2013). The levels of many seed-specific proteins including late embryo abundant (LEA) proteins, small heat shock proteins (sHSPs), and seed storage proteins are markedly reduced in *abi3* seeds (Parcy *et al.*, 1994; Wehmeyer *et al.*, 1996; Wehmeyer and Vierling, 2000). These genes are directly or indirectly regulated by ABI3, and such decreases in protein expression and accumulation may result in the pleiotropic phenotypes of the *abi3* mutants (Kotak *et al.*, 2007; Roschzttardtz *et al.*, 2009; Park *et al.*, 2011; Delmas *et al.*, 2013).

Recently it was shown that HvTIP3;1 plays a key role in preventing the coalescence of small PSVs in barley aleurone cells (Lee *et al.*, 2014). However, the function and physiological role of TIP3s in mature seeds are largely unknown. In the current work, the function of TIP3s in seed longevity was investigated. The data suggest that both TIP3 isoforms play roles in maintaining seed longevity and function under the control of ABI3.

## Materials and methods

### Plant materials and growth conditions

Col and L*er* were used as wild types (WTs) for the experiments, as indicated. The mutants *abi3-1* (CS24), *abi3-4* (CS6130), *fus3-3* (CS6128), *tip3;1* (SALK_053807.26.20), and *tip3;2* (SALK_125353C) were obtained from the *Arabidopsis* Biological Resource Center (ABRC). The *abi3-6* seeds were kindly provided by Dr Eiji Nambara. The homozygous seeds of *abi3-4*, *abi3-6*, and *fus3-3* were obtained by selecting green seeds. The T-DNA insertion sites in the *tip3;1* and *tip3;2* mutants were confirmed by PCR and sequencing analysis of the flanking regions. Homozygous plants were obtained and used in this study. The *tip3;1/tip3;2* double mutant was obtained by crossing the homozygotes of *tip3;1* and *tip3;2* mutants, and the double mutants were selected by PCR.

*Arabidopsis* seeds were surface sterilized for 20min in 10% bleach and washed five times with sterile water. Sterilized seeds were incubated for 48h at 4 °C in the dark, followed by germination on Murashige and Skoog (MS) medium containing MS salts, 10g l^–1^ sucrose, and 8g l^–1^ agar, pH 5.8. One-week-old seedlings were transferred to soil and grown in a growth chamber (22 °C, with a 16h light/8h dark photoperiod). To collect siliques at different developmental stages, blooming flowers were first marked by tying with cotton threads on the day of pollination. Mature seeds were harvested, dried, and stored at 20 °C.

### Plasmid construction and transgenic plants

Mutant *TIP3;1* promoters were generated by PCR-directed mutagenesis using a construct containing the 2kb *TIP3;1* promoter as a template (primers are listed in Supplementary Table S1 available at *JXB* online). WT and mutant promoters were cloned into the pCambia1300 plasmid and transformed into Col. Ten independent T_2_ transgenic lines per construct were randomly selected to determine the levels of β-glucuronidase (GUS) expression using real-time PCR (RT-PCR).

For the *TIP3;1*-RNAi (RNA interference) construct, the PDK intron product amplified from pKannibal was cloned into the PHB vector (Mao *et al.*, 2005) to generate the PHB-RNAi vector. Then, the 2kb *TIP3;1* promoter was cloned into the PHB-RNAi vector by replacing the 2×35S promoter to generate the PHB-Pro_*TIP3;1*_-RNAi vector. Then, the *TIP3;1* cDNA fragment was amplified and inserted in reverse orientation into both sides of the PDK intron. The PHB-Pro_*TIP3;1*_:*TIP3;1* RNAi plasmid was transformed into the *tip3;2* mutant background. RNAi transgenic lines (*TIP3;1*-RNAi/*tip3;2*) were obtained, and *TIP3;1* expression levels in T_3_ homozygous transgenic plants were analysed by RT-PCR and western blot analysis. T_4_ homozygous lines were used for germination and the controlled deterioration test (CDT). For the *Pro*_2×*35S*_:*TIP3;1* or *Pro*_2×*35S*_:*TIP3;2* construct, *TIP3;1* cDNA or *TIP3;2* cDNA was cloned into the multiple cloning site of the PHB vector.

### RNA extraction and quantitative RT-PCR analyses

Total RNA was isolated from dry mature seeds, siliques, imbibed seeds, and leaves using RNAiso for Polysaccharide-rich Plant Tissue (TaKaRa, Otsu, Shiga, Japan) according to the manufacturer’s instructions. Then, 1 μg of total RNA was reverse transcribed using a Primescript RT Reagent Kit with gDNA Eraser (TaKaRa). Quantitative RT-PCR (qRT-PCR) analyses were performed using the SYBR Green method (SYBR premix EX taq, TaKaRa) with the StepOnePlus™ Real-time PCR System (Applied Biosystems). The PCR program was as follows: 95 °C for 10 s, 60 °C for 35 s, repeated for 40 cycles.

To quantify gene expression in seed development and seed germination, geNorm 3.5 software was used to select four reference genes (*PP2A*, *CYP5*, *ACT7*, and *ACT8*) for seed development and another four reference genes (*PP2A*, *Ef1a*, *CYP5*, and *ACT8*) for seed germination from nine tested genes. The normalization factor, normalized GOI (gene of interest) quantity, and stand deviation (SD) of the normalized GOI quantity were calculated according to the geNorm manual (Vandesompele *et al.*, 2002).

### Protein extraction and immunoblot analyses

Proteins were extracted from *Arabidopsis* seeds with extraction buffer [0.1M TRIS-HCl pH 7.5, 0.15M NaCl, 20% glycerol, 5mM EDTA, 1% Triton X-100, 0.01M β-mercaptoethanol, 1mM phenylmethylsulphonyl fluoride (PMSF)] and denatured at 95 °C for 5min. Seed debris was removed by centrifugation at 12 000 *g* for 10min. Protein contents were determined using a Bradford assay. Then, 30 μg of protein was loaded onto a gel and separated by 12.5% SDS–PAGE. α-TIP polyclonal antibody was manufactured by ABclonal^®^ Technology (Wuhan, China), using sHQPLAPEDY peptide as antigen whose sequence was the same as previously reported (Jauh *et al.*, 1998). The γ-TIP antibody (Cat. no. AS09 493), HSP17.6 antibody (Cat. no. AS08 284), and HSP17.7 antibody (Cat. no. AS07 255) were purchased from Agrisera (Vännäs, Sweden) and the ACTIN antibody was purchased from Abmart (Shanghai, China). The horseradish peroxidase (HRP)-conjugated secondary antibody sc-2370 (Santa Cruz, Dallas, TX, USA) was used at a 1:10 000 dilution. Signals were detected using an ECL Detection Kit (Thermo Fisher Scientific, Waltham, MA, USA) and scanned with a ChemiDoc™ XRS+ Instrument (Bio-Rad, Hercules, CA, USA).

### Protoplast transformation and promoter activation assays

Protoplast isolation and transformation were performed according to the protocol described previoulsy (Yoo *et al.*, 2007; Wu *et al.*, 2009) with minor modifications. Protoplasts were isolated from rosette leaves of 4-week-old *Arabidopsis* plants using the tape method as described (Wu *et al.*, 2009).

Promoter activation assays were performed with a dual-luciferase reporter assay system (Hellens *et al.*, 2005). *ABI3* and *FUS3* cDNAs were cloned into the effector plasmid pGreenII 62-SK, and *TIP3;1* and *TIP3;2* promoter sequences were cloned into the reporter plasmid pGreenII 0800-LUC, respectively. For transfection, a plasmid mixture (12 μg of effector plasmids and 4 μg of dual-luciferase reporter plasmids) was added to 100 μl of protoplasts (~2×10^4^ cells). The transfected protoplasts were incubated in the dark for >16h in the presence or absence of 5 μM ABA. Dual-luciferases activity was assayed using Dual Luciferase Assay Reagents (Promega, Madison, WI, USA) according to the manufacturer’s instructions and measured with a Varioska Flash spectral scanning multimode reader (Thermo Fisher). For immunoblotting, 50 μg of effector plasmids were added to 500 μl of protoplasts (~10^5^ cells) in the presence of 5 μM ABA.

### Recombinant protein purification and EMSA

A partial *ABI3* fragment (encoding R559-K720) including the B3 domain was cloned into pET28a (Novagen) and transformed into *Escherichia coli*. The recombinant protein was induced at 16 °C and purified in its native form using Ni-NTA agarose (Qiagen, Venlo, Limburg, The Netherlands) following the manufacturer’s protocol.

Elecrophoretic mobility shift assays (EMSAs) were performed using a LightShift Chemiluminescent EMSA Kit (Thermo Fisher). The B3 domain of recombinant ABI3 protein was incubated with biotin-labelled probes containing different RY motif fragments at 20 °C for 30min in the binding system [1× binding buffer, 2.5% glycerol, 50ng μl^–1^ poly(dI–dC), 50mM KCl, and 0.5mM EDTA]. DNA–protein complexes were separated by 6% TRIS/borate/EDTA PAGE and transferred onto a Hybond-N^+^ nylon membrane (GE Healthcare Life Sciences, USA). Biotin-labelled probes were detected by HRP-conjugated streptavidin and visualized with an ECL Detection Kit according to the manufacturer’s instructions.

### Yeast one-hybrid and DNA–protein interaction ELISA

Yeast one-hybrid assays were performed using a Matchmaker One-hybrid system (Clontech, Mountain View, CA, USA). Three tandem copies of the RY2 element of Pro_*TIP3;1*_ (GGCACACATGCATGCTTAGT) and three copies of the RY element of Pro_*TIP3;2*_ (CTTGGCACACATGCATAGATATAT) were cloned upstream of the *HIS3* reporter gene in the pHISi vector, respectively. These reporter constructs, as well as the empty pHISi vector, were linearized with *Xba*I and integrated into the genome of the YM4271 strain to generate reporter strains. The reporter strains were transformed again with pGAD424-*ABI3* (559R-720K) or empty vector pGAD424, respectively. Yeast transformants were grown on synthetic SD-Leu-His medium, and binding activity was monitored on SD-Leu-His medium supplemented with 30mM or 60mM 3-amino-1,2,4-triazole (3-AT).

DNA–protein interaction-enzyme-linked immunosorbent assay (DPI-ELISA) was performed as described in Brand *et al.* (2010). Full-length glutathione *S*-transferase (GST)–ABI3 protein was produced in the BL21-Codon Plus strain and purified using Glutathione Superflow Resin (Qiagen). An antibody against GST conjugated with HRP was used to detect the bound proteins.

### Controlled deterioration test and basal thermotolerance assay

The CDT was performed as described previously with minor modifications (Tesnier *et al.*, 2002; Oge *et al.*, 2008; Chen *et al.*, 2012). Different *Arabidopsis* seeds for the test were harvested from plants at the same time, dried, and stored under the same conditions (20 °C in a desiccator containing blue self-indicating silica gel) for at least 2 weeks prior to the experiment unless otherwise indicated. The seeds were equilibrated for 3 d at 15 °C and 85% relative humidity (RH). After equilibration, the seeds were stored at 40 °C and 80% RH in a temperature- and humidity-controlled incubator. The temperature and RH in the incubator were corrected and monitored using a thermohygrometer (Testo 608-H1, Germany). The seeds were stored under these conditions for 1–7 d. After storage at high temperature and RH, the seeds were stored at 20 °C and 33% RH for 3 d and dried to 6% moisture content. Seed moisture content was determined by weighing the seeds before and after drying at 105 °C for 24h.

The basal thermotolerance assay (BTA) was performed as described (Prieto-Dapena *et al.*, 2006). Seeds used for testing were harvested at the same time, dried, and stored under the same conditions for at least 2 weeks prior to the experiment. The seeds were imbibed in Eppendorf tubes and incubated at 50 °C for 1–4h. After treatment, the seeds were cooled to room temperature and plated on MS medium. For HgCl_2_ treatment, imbibed seeds were incubated at 42 °C for various times in the presence of 50 μM HgCl_2_ or 1mM dithiothreitol (DTT), washed five times in sterile water, and then grown on MS medium.

All germination analyses were performed with four replicates using ~100 seeds per replicate. The germination percentage was calculated as the mean ±SD.

### Tetrazolium assay and H_2_O_2_ staining

The tetrazolium assay was performed as described by Wharton (1955) with minor modifications. Embryos isolated from imbibed seeds after the CDT were soaked in a solution of 1% 2,3,5-triphenyl tetrazolium chloride (Sigma-Aldrich) and incubated at 22 °C for 12h. Viable embryos stained red, and non-viable or dead embryos remained unstained. Seed viability was evaluated by examining the staining pattern and colour intensity.

For H_2_O_2_ staining, isolated embryos were stained with 1mg ml^–1^ 3,3′-diaminobenzidine (DAB; Sigma-Aldrich) solution. The embryos were incubated in DAB solution at 22 °C for 8h. After staining, the embryos were bleached with 95% ethanol.

### Hydrogen peroxide permeability assay in yeast

Yeast strains *Δdur3* (MATa, *ura3*), *Δyap1* (MATa, *ura3*), and *Δskn7* (MATa, *ura3*) were transformed with pYX212 (or derivatives of pYX212 carrying AQP cDNAs). Yeast transformants were inoculated and grown to the stationary phase. The cells were diluted to an OD_600_ of 1.0 with SD-Ura liquid medium. A 10 μl aliquot of 0.1 OD cells was spotted onto SD-Ura medium containing the indicated concentration of H_2_O_2_. Photographs were taken 3 d after incubation at 30 °C.

For the fluorescence assays, WT (THY.AP4) cells and pYX212 or AQP transformants were grown to mid logarithmic phase, incubated with 30 μM CM-H_2_DCFDA (Molecular Probes, Life Technologies) for 45min, washed five times in 20mM HEPES (pH 7.0), and finally suspended in HEPES buffer at an OD_600_ value 1.0. Fluorescence was measured with the Varioska Flash spectral scanning multimode reader at excitation/emission of 492/527nm and a temperature of 20 °C.

### Hypo-osmotic yeast protoplast swelling assay

THY.AP4 yeast cells transformed with pYX212 or derivatives of pYX212 carrying AQP cDNAs were grown to mid logarithmic phase, harvested, washed twice in sterile water, and suspended in SCE buffer (1M sorbitol, 0.1M sodium citrate, 10mM EDTA, 0.2mM β-mercaptoethanol, pH 6.8) containing 200U ml^–1^ lyticase (L2524, Sigma-Aldrich) for 2h at 30 °C. Following centrifugation, protoplasts were washed twice and resuspended in STC buffer (1M sorbitol, 10mM TRIS-HCl pH 7.5, 10mM CaCl_2_) to the same OD_600_. Protoplasts were diluted to 0.5M sorbitol with sterile water using a syringe dispenser. The change of OD_600_ value was monitored per 0.1 s with the Varioska Flash spectral scanning multimode reader in a flash mode.

## Results

### TIP3s are specifically expressed during seed maturation

**Fig. 1.**
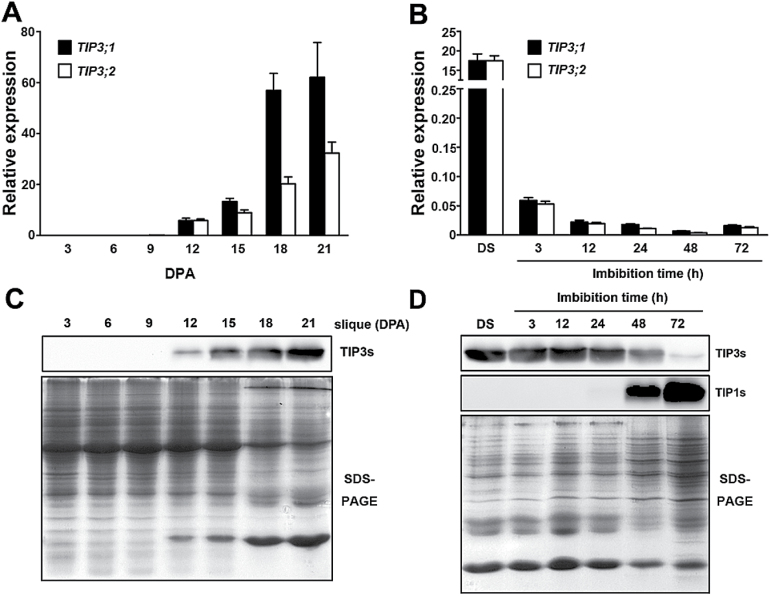
*TIP3* genes are specifically expressed during seed maturation. (A and B) Expression analysis of *TIP3;1* and *TIP3;2* during seed development (A) and seed germination (B) in *Arabidopsis*. qRT-PCR analysis of *TIP3;1* and *TIP3;2* transcript abundance during seed development and seed germination. The relative expression level of each gene was normalized with four reference genes, and calculated according to the geNorm 3.5 manual. Values are means ±SD, *n*=3. DPA, days post-anthesis. (C and D) Immunoblot analysis of TIP3s during seed development (C) and seed germination (D). The same amounts of proteins separated by SDS–PAGE were stained with Coomassie Brilliant Blue and used as a loading control.

The *Arabidopsis* genome contains two *TIP3* genes, namely *TIP3;1* and *TIP3;2*. Using qRT-PCR, the temporal expression patterns of *TIP3* genes were investigated in a precise manner. Transcripts of *TIP3;1* and *TIP3;2* began to be detectable in siliques at 12 days post-anthesis (DPA) (Fig. 1A). *TIP3* transcript levels increased sharply throughout the maturation phase. Immunoblot analysis also indicated that TIP3s began to accumulate at the same time point (Fig. 1C). Since the antibody raised against the C-terminal peptide of TIP3;1 cannot discriminate between TIP3;1 and TP3;2 (Supplementary Fig. S1 at *JXB* online), the detected signals represented both TIP3 isoforms. In germinating seeds, the levels of *TIP3* transcripts decreased to <1% during the first 3h after germination (Fig. 1B). Interestingly, the protein levels of TIP3s did not decrease significantly within 24h, but they started to decrease sharply 48h after germination (Fig. 1D). At the same time, TIP1s (TIP1;1 and TIP1;2) began to be detectable (Fig. 1D). Fluorescent signals were detected in the seeds of *Pro*_*TIP3;1*_ *:GFP* transgenic plants, but not in WT seeds (Supplementary Fig. S2). These data suggest that *TIP3;1* and *TIP3;2* are specifically expressed in seeds, and the *TIP3;1* promoter is active in seeds.

### TIP3;1 and TIP3;2 are required for seed longevity

**Fig. 2.**
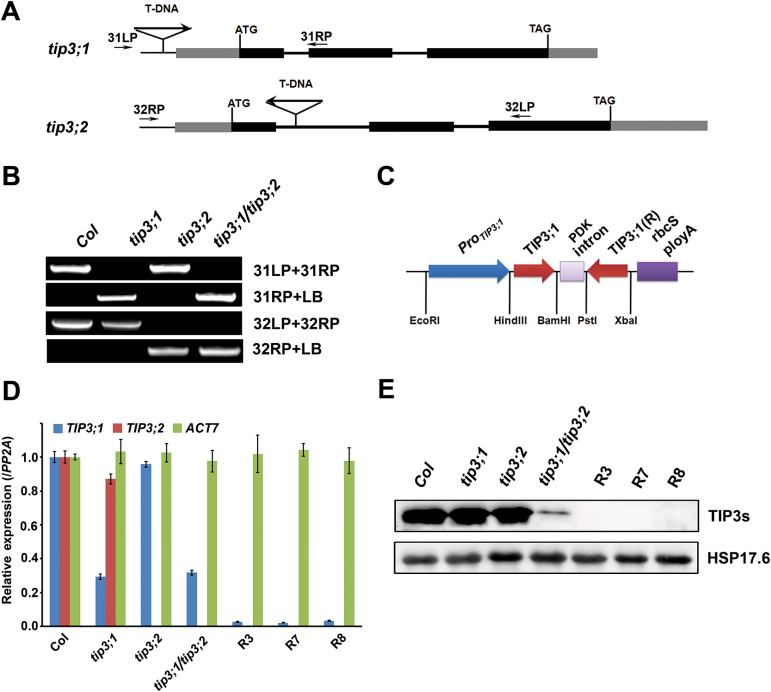
Identification of *tip3;1* and *tip3;2* T-DNA insertion mutants and three *TIP3;1*-RNAi transgenic lines (*TIP3;1*-RNAi/*tip3;2*) in the *tip3;2* mutant background. (A) Schematic representation of the *tip3;1* and *tip3;2* T-DNA insertion mutant lines. A triangle indicates the position of the T-DNA insertion, and the arrow indicates its orientation. The genomic sequences corresponding to the coding region (black boxes), untranslated region (grey boxes), and introns (black lines) are indicated. The positions of the primers (31LP, 31RP, 32LP, and 32RP) used for PCR analysis of the *tip3;1* and *tip3;2* T-DNA insertion mutants, respectively, are also indicated. (B) PCR analysis of genomic DNA of Col, *tip3;1*, *tip3;2*, and *tip3;1/tip3;2*. LP, left primer; RP, right primer; LB, T-DNA left border primer. (C) Schematic representation of the construct used for the suppression of *TIP3;1* in *Arabidopsis* seeds. RNAi technology was used with a segment of the *TIP3;1* gene driven by the seed-specific *TIP3;1* promoter. (D) qRT-PCR analysis of *TIP3;1*, *TIP3;2*, and *ACT7* transcript abundance in mature seeds of Col, mutants, and RNAi lines. *PP2A* was used as the endogenous control, and the transcript abundance of *TIP3;1*, *TIP3;2*, and *ACT7* was quantified by comparisons with that of *PP2A*. Values are means ±SD, *n*=3. (E) Immunoblot analysis of TIP3s in mature seeds from Col, *tip3* mutants, and three *TIP3;1*-RNAi/*tip3;2* transgenic lines (R3, R7, and R8). HSP17.6, which is expressed in mature seeds, was used as a loading control. (This figure is available in colour at *JXB* online.)

To characterize the effects of loss of function of *TIP3* genes, two T-DNA insertion mutants were obtained from the ABRC. PCR analysis of genomic DNA from the mutants confirmed the locations of the T-DNA insertions. SALK_053807 has an insertion in the promoter region between the RY2 and RY3 motif of *TIP3;1*, and SALK_125353c has an insertion in the first intron of *TIP3;2* (Fig. 2A, B). The expression level of *TIP3;1* in *tip3;1* seeds was reduced to 30% of that in WT seeds (Fig. 2D). *TIP3;2* transcripts were not detectable in *tip3;2* seeds (Fig. 2D), demonstrating that the *tip3;2* mutant is transcript null.

Plants of both mutants are phenotypically indistinguishable from WT plants under normal growth conditions (Supplementary Fig. S3 at *JXB* online). To verify whether *TIP3;1* and *TIP3;2* are redundant genes, a *tip3;1/tip3;2* double mutant was generated (Fig. 2B). Since *tip3;1* is not a null mutant, *TIP3;1* was expressed at low levels in double mutant seeds (Fig. 2D). RNAi was used to reduce the level of *TIP3;1* expression in the *tip3;2* mutant background (Fig. 2C). Three homozygous T_3_ transgenic lines, R3, R7, and R8, were obtained. The expression of *TIP3;1* was significantly reduced in all three RNAi lines (Fig. 2D). Immunoblot analysis showed that the levels of TIP3;1 were much lower in *TIP3;1*-RNAi/*tip3;2* (R3, R7, and R8) transgenic seeds than in *tip3;1/tip3;2* double mutant seeds (Fig. 2E).

**Fig. 3.**
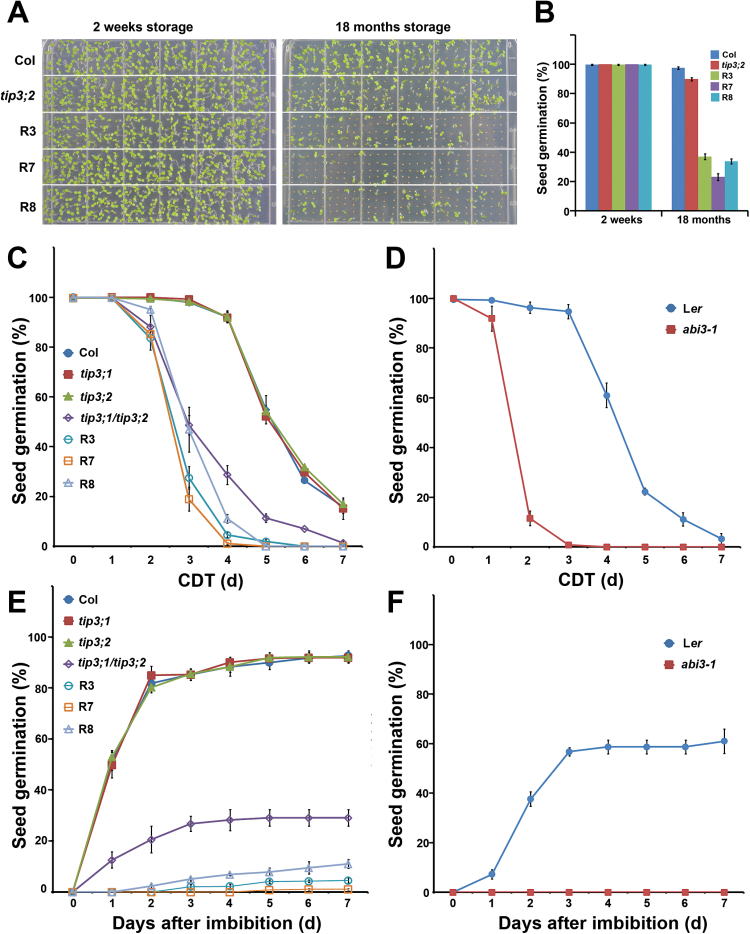
Natural and artificial seed ageing assays showing that *TIP3* genes are involved in maintaining seed longevity. (A) Germination and growth of seeds from Col, *tip3;2*, and three lines of *TIP3;1*-RNAi/*tip3;2* stored for 2 weeks or 18 months. The photographs were taken 6 d after seed imbibition. (B) Germination percentages of seeds 6 d after imbibition. (C) Germination percentages of *tip3* mutants and *TIP3;1*-RNAi/*tip3;2* transgenic seeds submitted to a CDT for 1–7 d. The germination percentages were counted 7 d after imbibition. (D) Germination percentages of L*er* and *abi3-1* seeds submitted to a CDT for 1–7 d. The germination percentages were counted 7 d after imbibition. (E) Germination percentages of *tip3* mutants and *TIP3;1*-RNAi/*tip3;2* transgenic seeds after a 4 d CDT. Germination percentages were counted at different time points after imbibition. (F) Germination percentages of L*er* and *abi3-1* seeds after a 4 d CDT. Germination percentages were counted at different time points after imbibition. Values are the means ±SD of four technical replicates with 100 seeds per replicate. In (C–E) seeds used for germination tests were harvested at the same time and stored for 2 weeks prior to the experiment.

The seed germination percentage of *TIP3;1*-RNAi/*tip3;2* plants was tested. No significant difference was observed between *TIP3;1*-RNAi/*tip3;2* and WT seeds which were stored for 2 weeks after harvesting (Fig. 3A). Comparing the germination percentage for 18-month-old seeds, WT and *tip3;2* seeds remained at 98% and 95%, respectively. In contrast, the germination percentage of *TIP3;1*-RNAi/*tip3;2* seeds decreased to <40% (Fig. 3A, B).

This observation prompted the study of whether seed longevity was affected by the null function of the *TIP3* genes. BTAs, which partially reflect seed longevity and viability, were first performed. As a general inhibitor of AQPs, mercury binds to the cysteine residue near the pore site and inhibits the channel activity of AQP by occluding the pore (Daniels *et al.*, 1996; Maurel and Chrispeels, 2001; Savage and Stroud, 2007). DTT can function as a scavenger to reverse the inhibition effect of mercury (Martre *et al.*, 2001; Vander Willigen *et al.*, 2006). When seeds were incubated for 24h at 22 °C in the presence of 50 μM HgCl_2_, seed viability was not impaired. However, the seed germination percentage decreased when incubated at 42 °C in the presence of HgCl_2_ (Supplementary Fig. S4A at *JXB* online). The effect was partially reversed by adding 1mM DTT, suggesting that the activities of AQPs in seeds may be involved in seed longevity.

BTAs were then performed with *tip3* mutant seeds. Imbibed seeds were directly incubated at 50 °C for various periods of time. After 1h of treatment, the germination percentage of *tip3;1/tip3;2* double mutant seeds declined drastically compared with WT, *tip3;1*, and *tip3;2* seeds (Supplementary Fig. S4B at *JXB* online). After 2h of treatment, the germination percentages of WT, *tip3;1*, and *tip3;2* seeds decreased significantly but were still much higher than that of the double mutant seeds (Supplementary Fig. S4B). Therefore, *tip3;1/tip3;2* double mutant seeds are more sensitive to heat stress than WT and single mutants.

**Fig. 4.**
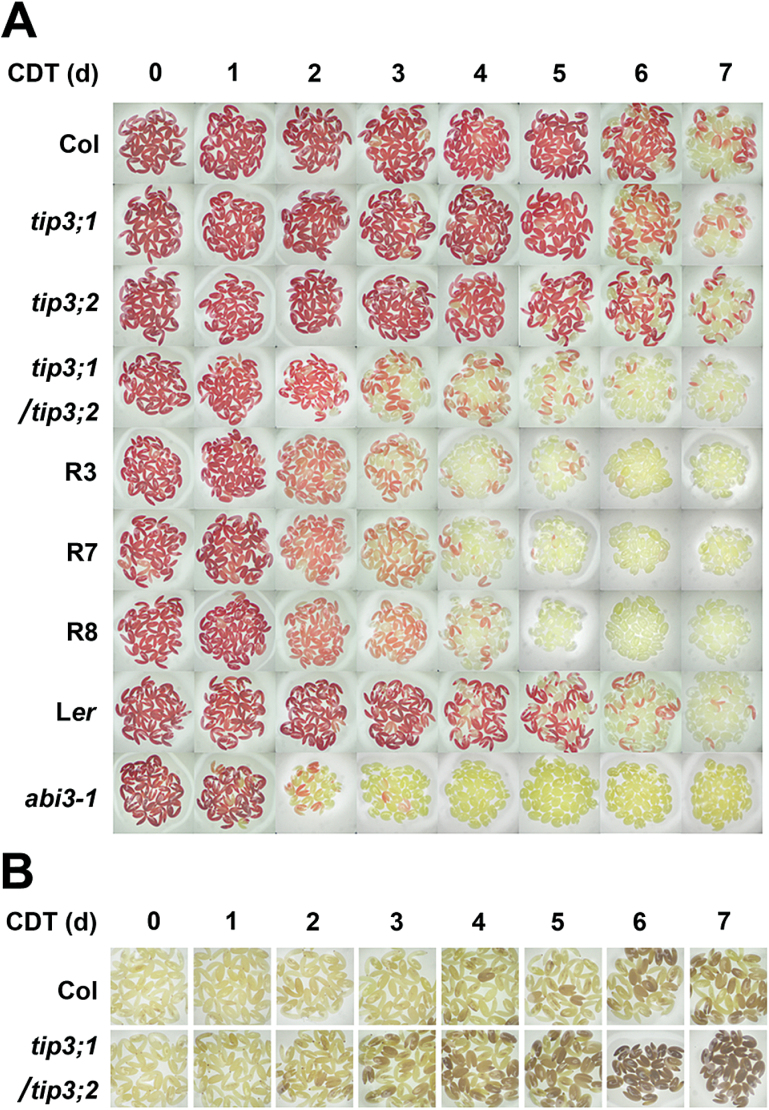
TIP3s are involved in maintaining seed viability during the CDT. (A) Seed viability after a 0–7 d CDT. Seed viability was analysed by tetrazolium staining. (B) Staining of H_2_O_2_ in the embryos of Col and *tip3;1/tip3;2* seeds submitted to a CDT for 0–7 d. Seeds used for the CDT were harvested at the same time and stored for 2 weeks prior to the assay.

Seed longevity was further estimated using the CDT. The CDT accelerates seed ageing by increasing the temperature of seed storage and the seed moisture content. The germination percentage of all untreated seeds was ~100% at 7 d after germination. When treated at 40 °C with 80% RH for 4 d, WT, *tip3;1*, and *tip3;2* seeds had a germination percentage of ~90%, whereas that of *tip3;1/tip3;2* double mutant seeds was only 30% (Fig. 3C, E). Furthermore, the germination percentages of the seeds of three *TIP3;1*-RNAi/*tip3;2* transgenic lines were further reduced to 1–10% (Fig. 3C, E). The seed germination percentage was <50% for *tip3;1/tip3;2* and *TIP3;1*-RNAi/*tip3;2* seeds after 3 d of the CDT, whereas for the *tip3;1*, *tip3;2*, and WT seeds, the germination percentage was <50% after 5 d of the CDT (Fig. 3C). Unlike the severe alleles of the *ABI3* mutant, *abi3-1* and *abi3-7* seeds are desiccation tolerant but with decreased longevity (Ooms *et al.*, 1993; Bies-Etheve *et al.*, 1999; Tesnier *et al.*, 2002). *abi3-1* mutant seeds were also subjected to the CDT. The seed germination percentage decreased to 10% at 2 d after the CDT and to <1% after 3 d (Fig. 3D), which was even lower than that of *tip3* knockdown mutant seeds. At 4 d after the CDT, *abi3-1* seeds cannot germinate (Fig. 3F). A tetrazolium assay confirmed that the seeds of the *tip3;1/tip3;2* double mutant and three *TIP3;1*-RNAi/*tip3;2* transgenic lines began to lose viability 3 d after the CDT, which was earlier than observed in the WT, *tip3;1*, and *tip3;2* mutants (Fig. 4A). Again, the *abi3-1* mutant was the most sensitive to the CDT, as its seeds started to lose viability after 2 d. Taken together, these results show that TIP3s are required for seed longevity.

### Knockdown of TIP3;1 and TIP3;2 results in the elevated accumulation of hydrogen peroxide upon CDT

One of the most critical factors that influence seed ageing is the accumulation of reactive oxygen species (ROS) in seeds (Bailly, 2004). ROS lead to lipid peroxidation, DNA damage, and inactivation of enzymes. The genes encoding proteins which can scavenge ROS or repair DNA damage and protect proteins were reported to be involved in seed longevity and seed viability (Sattler *et al.*, 2004; Oge *et al.*, 2008; Waterworth *et al.*, 2010; Chen *et al.*, 2012; Verma *et al.*, 2013; Wang *et al.*, 2014). DAB staining showed that the H_2_O_2_ contents in seeds increased during the CDT and accumulated to higher levels in *tip3;1/tip3;2* seeds than in WT seeds (Fig. 4B).

### TIP3;1 and TIP3;2 are activated by ABI3 during seed maturation

**Fig. 5.**
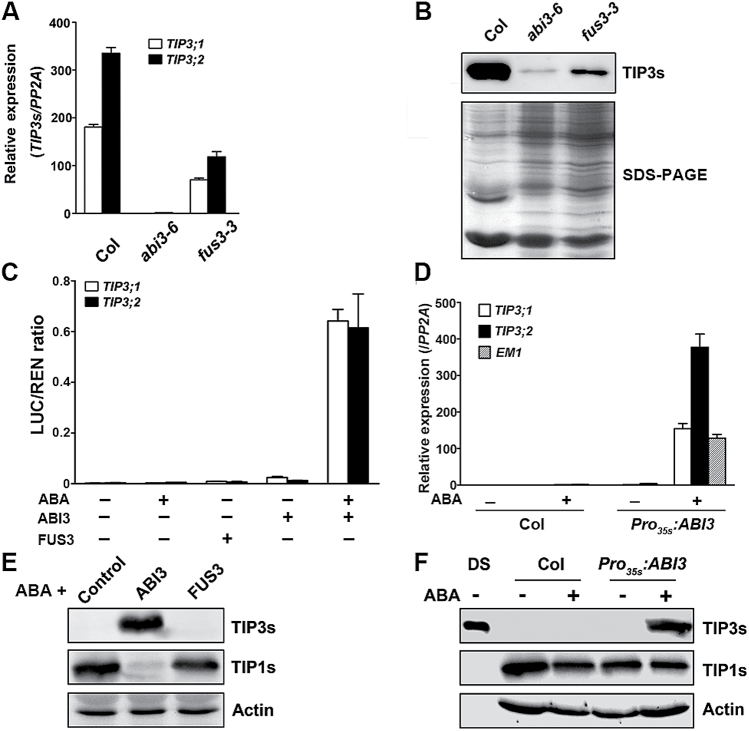
ABI3 regulates the expression of *TIP3* genes. (A and B) qRT-PCR and immunoblot analysis of the expression of *TIP3* genes in *abi3-6* and *fus3-3* seeds. Values in (A) are means ±SD, *n*=3. (C) The *TIP3;1* and *TIP3;2* promoters are activated by ABI3 when treated with ABA in a transient expression assay. Values are means ±SD, *n*=3. Protoplasts transformed with empty pGREENII 62-SK vector were used as a control; 5 μM ABA was supplied in the ABA treatment. (D) qRT-PCR analysis of *TIP3* and *EM1* transcript levels in the WT (Col) and a transgenic line ectopically expressing ABI3 (*Pro*_*35S*_ *:ABI3*). For ABA treatment, 3-week-old seedlings grown on MS medium were transferred to MS medium supplemented with 50 μM ABA for 3 d. *PP2A* was used as an endogenous control. (E) Immunoblot analysis of TIP3s and TIP1s in protoplasts transiently expressing *ABI3* or *FUS3* in the presence of 5 μM ABA. Detection of actin by an antibody was used as a loading control. (F) Immunoblot analysis of TIP3s in seedlings of WT and *Pro*_*35S*_ *:ABI3* transgenic *Arabidopsis*. ABA treatment was performed as described in (D). DS, dry mature seeds.

The transcription factor ABI3 is involved in seed desiccation tolerance and seed longevity (Ooms *et al.*, 1993; Tesnier *et al.*, 2002). *TIP3* genes are seed-specific genes during seed maturation, and *tip3* knockdown mutant seeds exhibit a decrease of seed longevity similar to the seed longevity phenotype of *abi3-1* and *abi3-7.* It was hypothesized that TIP3s may maintain seed longevity under the expressional regulation of ABI3. To test whether the seed-specific transcription factors ABI3 or FUS3 are involved in the regulation of *TIP3* gene expression in seeds, the presence of *TIP3* gene transcripts in the corresponding mutant seeds was investigated. As expected, *TIP3;1* and *TIP3;2* transcripts were not detectable in *abi3-6* mutant seeds (Fig. 5A). The *abi3-6* allele contains a deletion in *ABI3* which causes a premature stop codon and leads to translation of a short form protein containing only the A1 domain but not the B1, B2, and B3 domains. The expression levels of *TIP3* genes in the *fus3-3* mutant decreased ~50% compared with those of the WT (Col) (Fig. 5A). The protein levels of TIP3s decreased significantly in the *abi3-6* and *fus3-3* mutants (Fig. 5B). The expression levels of *ABI3* in *abi3-6* and *fus3-3* mutant seeds were also analysed. In *fus3-3* seeds, *ABI3* expression decreased to 50% compared with the WT (Supplementary Fig. S5 at *JXB* online). Therefore, the reduction of *TIP3* gene expression in *fus3-3* correlates with decreased expression of *ABI3* in *fus3-3* mutant seeds. the expression of *TIP3* genes was also analysed in other alleles of ABI3, namely *abi3-1*, *abi3-4*, and *abi3-8*. The amount of *TIP3* transcripts and the abundance of proteins were reduced to varying degrees in these *abi3* mutants (Supplementary Fig. S6). *TIP3;1* promoter activity was also reduced in the *abi3-6* mutant. No green fluorescent protein (GFP) fluorescence and GFP protein expression could be detected in isolated *abi3-6* embryos transformed with *Pro*_*TIP3;1*_ *:GFP* (Supplementary Fig. S7). Taken together, these results suggest that ABI3 is required for *TIP3* gene expression and protein accumulation in mature seeds.

Compared with the empty vector, transient expression of *ABI3* in the protoplasts slightly increased the activity of the *TIP3;1* and *TIP3;2* promoters (Fig. 5C). ABA alone could not activate *TIP3* promoters, but addition of ABA to *ABI3*-expressing protoplasts caused drastic induction of *TIP3;1* and *TIP3;2* promoter activity by 279- and 150-fold, respectively, as indicated by the LUC/REN ratio (Fig. 5C). TIP3 proteins accumulated in ABA-treated protoplasts expressing *ABI3*, but not in the protoplasts expressing *FUS3* (Fig. 5E). Consistent with other seed-specific genes (Parcy *et al.*, 1994), ectopic expression of *ABI3* also led to the accumulation of *TIP3* transcripts as well as TIP3 proteins in vegetative tissues only when treated with ABA (Fig. 5D, F). These results suggest that ABI3 can activate the expression of *TIP3* genes in the presence of ABA and is a transcriptional regulator of *TIP3* genes.

### ABI3 binds to the RY motifs of TIP3 promoters

**Fig. 6.**
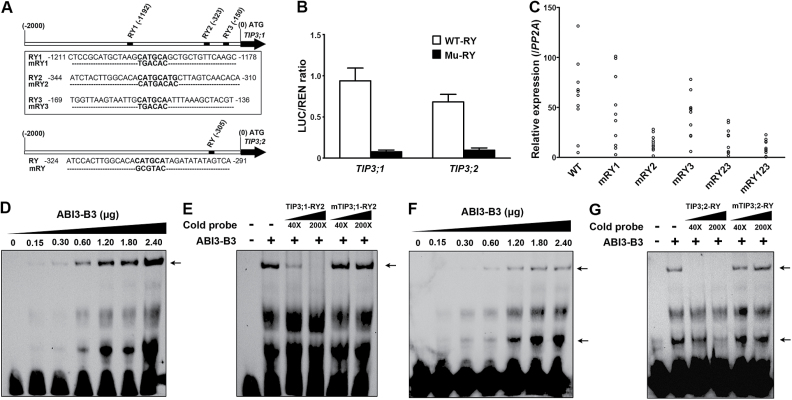
ABI3 binds to the *TIP3* promoters through their RY motifs. (A) Diagram of the *TIP3;1* and *TIP3;2* promoter regions. The RY motifs are shown in black boxes. (B) Transient expression assay with mutant *TIP3;1* and *TIP3;2* promoters. The *TIP3;1* mutant promoter contains mutations in the RY1, RY2, and RY3 motifs. The *TIP3;2* mutant promoter contains a mutation in the RY motif. Protoplasts were transformed with the effector plasmid containing *ABI3* and treated with 5 μM ABA. Values are means ±SD, *n*=3. (C) Relative expression levels of the *GUS* reporter gene driven by the *TIP3;1* promoters with or without mutations in the RY motifs. RNA was extracted from seeds of 10 independent transgenic lines carrying the WT or mutant promoters fused to *GUS*. Each point represents the mean of three replicates of one transgenic line, and SD values were omitted for clarity. (D and F) EMSA demonstrating the binding of the B3 domain of ABI3 to the RY2 element in the *TIP3;1* promoter (D) or the RY element in the *TIP3;2* promoter (F). The numbers indicate the amount of B3 domain of ABI3 protein used in the assays. (E and G) Binding specificity of ABI3 protein to the RY2 element in the *TIP3;1* promoter (E) and the RY element in the *TIP3;2* promoter (G). Binding specificity was demonstrated with competition experiments by adding 40- or 200-fold excessive non-labelled WT or mutant probes. Arrows indicate the gel retardation complexes formed between RY elements and the B3 domain of ABI3 protein.

The B3 domain of ABI3 has DNA binding specificity and recognizes the RY motif (CATGCA) (Suzuki *et al.*, 1997). Promoter sequence analysis showed that *TIP3;1* and *TIP3;2* promoters possess three and one potential RY motif, respectively (Fig. 6A). To determine whether RY motifs are critical for the activation of *TIP3* promoters by ABI3, transient expression assays were performed with the *TIP3;1* promoter mutated in RY1, RY2, and RY3 motifs and the *TIP3;2* promoter mutated in the RY motif, respectively. Mutations in RY motifs caused reduction of both *TIP3* promoter activities (Fig. 6B), suggesting that the RY motifs are required for the promoter activities of *TIP3* genes. To determine further which RY motif is important for *TIP3;1* promoter activity, promoters containing mutations in the RY motif were fused to the *GUS* reporter gene and transgenic *Arabidopsis* plants were generated. *GUS* expression in transgenic seeds of different lines was determined by qRT-PCR. Mutation of the RY3 or RY1 motif caused a slight reduction in *GUS* expression, while mutation of the RY2 motif caused a more significant reduction in *GUS* expression (Fig. 6C). Additional mutations in RY3 or in the RY1 and RY3 motifs did not further reduce the activities of promoters containing a mutation in the RY2 motif (Fig. 6C). This result suggests that the RY2 motif is essential for *TIP3;1* expression in seeds.

The EMSA was performed to test whether ABI3 directly binds to the RY2 and RY motifs in the *TIP3;1* and *TIP3;2* promoters, respectively. The retarded protein–nucleotide complexes were detected in the presence of the B3 domain of ABI3 protein and biotin-labelled RY motifs from *TIP3;1* and *TIP3;2* promoters. The binding activity increased with increasing concentration of ABI3-B3 protein (Fig. 6D, F). When unlabelled *TIP3;1* or *TIP3;2* probes were added to the system as competitors, the levels of retarded complexes decreased (Fig. 6E, G). Additionally, ABI3-B3 did not bind to unlabelled *TIP3;1* or *TIP3;2* probes harbouring mutations in the RY motifs, and the levels of retarded complex did not decrease. Yeast one-hybrid assays and DPI- ELISA also showed that ABI3 binds RY elements from *TIP3* promoters (Supplementary Fig. S8 at *JXB* online).

### TIP3;2 facilitates both water and hydrogen peroxide diffusion

In addition to transporting water, some TIPs and plasma membrane intrinsic proteins (PIPs) facilitate H_2_O_2_ diffusion across the membrane (Bienert *et al.*, 2007, 2014; Dynowski *et al.*, 2008; Hooijmaijers *et al.*, 2012). In order to understand the connection between TIP3 transport activity and biological function, the water and H_2_O_2_ permeability of TIP3s was analysed. To test whether TIP3;1 and TIP3;2 have water channel activity, hypo-osmotic yeast protoplast swelling assays were performed. Hypo-osmotic shock causes water influx and bursting of yeast protoplasts, which could be monitored by a decrease at OD_600_. The yeast protoplasts expressing *TIP3;1* or *TIP3;2* burst much more quickly than protoplasts transformed with empty plasmid (Supplementary Fig. S9 at *JXB* online), suggesting that both TIP3;1 and TIP3;2 have water channel activity.

**Fig. 7.**
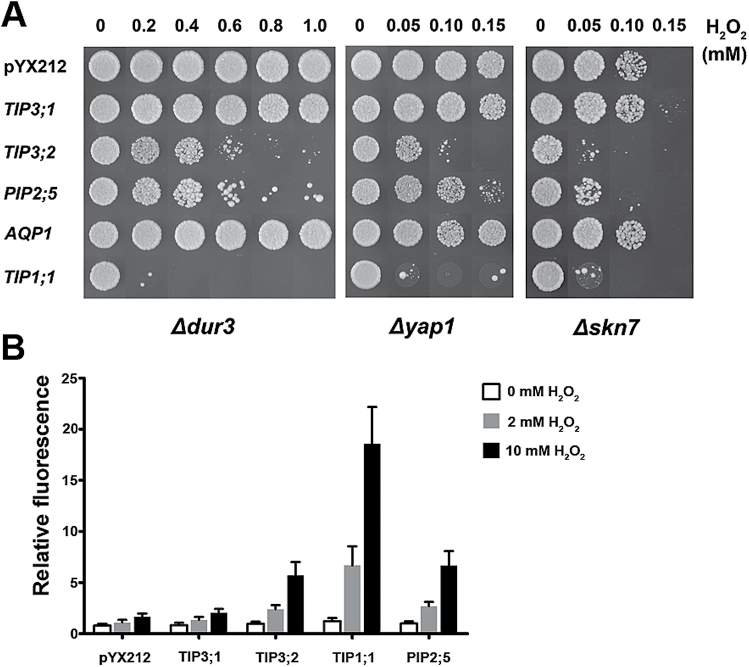
TIP3;2 facilitates H_2_O_2_ diffusion. (A) Survival test of three different yeast strains transformed with *TIP3* genes on medium containing H_2_O_2_. Yeast strains *Δdur3*, *Δyap1*, and *Δskn7* were transformed with pYX212 (or derivatives of pYX212 carrying AQP cDNAs). Yeast cells were diluted to an OD_600_ of 0.1 with SD-Ura liquid medium, and 10 μl were spotted onto SD-Ura medium containing various concentrations of H_2_O_2_. Numbers indicate the concentration of H_2_O_2_ (mM). Photographs were taken 3 d after incubation at 30 °C. (B) TIP3;2 mediates H_2_O_2_ diffusion across the membrane in yeast. The fluorescence of CM-H_2_DCFDA-loaded yeast cells transformed with pYX212 or pYX212 carrying the indicated *AQP* cDNAs wase measured 30min after incubation with 0, 2, or 10mM H_2_O_2_. Histograms represent the average increase in fluorescence after 30min incubation. Data are means ±SD, *n*=3.

To determine whether TIP3;1 and/or TIP3;2 is permeable to H_2_O_2_, yeast cells transformed with *TIP3* cDNAs were grown on synthetic medium containing different concentrations of H_2_O_2_. TIP1;1 and PIP2;5, which can facilitate H_2_O_2_ diffusion across the membrane, were used as positive controls. Three yeast strains differing in H_2_O_2_ sensitivity, namely *Δdur3*, *Δyap1*, and *Δskn7*, were used. The result of the growth test showed that the expression of *TIP3;2* significantly reduced cell growth and survival on medium containing H_2_O_2_ (Fig. 7A). When *TIP3;1* was expressed in yeast, the growth of yeast cells was not significantly changed in the presence of H_2_O_2_ compared with the negative controls.

TIP3-mediated uptake of H_2_O_2_ was further confirmed by using CM-H_2_DCFDA, a dye which was used to measure the ROS level in living cells. Upon exposure to H_2_O_2_, the intracellular level of accumulated ROS was higher in *TIP3;2* transformants compared with cells transformed with the empty vector (Fig. 7B). TIPl;1 showed higher H_2_O_2_ permeability than TIP3;2 and PIP2;5. *TIP3;1* yeast transformants showed almost the same increase in the intracellular level of ROS compared with cells transformed with the empty vector (Fig. 7B). These results indicate that TIP3;2 but not TIP3;1 can facilitate H_2_O_2_ permeation.

## Discussion

### TIP3 proteins are involved in maintaining seed longevity and contribute to the ABI3-controlled seed longevity pathway

The AQP family is highly diverse in higher plants and is represented by >30 members in one plant species. Therefore, AQPs belonging to the same group may have functional redundancy (Li *et al.*, 2013). This observation may explain why only a few plant AQP mutants possess clear phenotypes. The protein sequence of TIP3;1 shares 85% identity with that of TIP3;2, suggesting that these two proteins may be functionally redundant. Indeed, only the *tip3;1/tip3;2* double mutant (and not the *tip3;1* or *tip3;2* single mutant) exhibited a significant difference in seed longevity compared with the WT. Seeds of the *tip3;1/tip3;2* double mutant were more sensitive to prolonged storage and artificial ageing than the WT (Figs 3, 4). The abundance of TIP3s in seeds appears to be positively correlated with seed longevity, as the *TIP3;1*-RNAi/*tip3;2* lines were more sensitive to the CDT than the *tip3;1/tip3;2* mutant, which exhibits higher levels of TIP3;1 (Figs 2E, 3, 4).

Seed development can be divided into several phases, namely embryogenesis, seed filling, late maturation, and pod abscission. Two important traits of seeds, desiccation tolerance (i.e. ‘the ability to survive complete drying and rehydration’) and seed longevity (i.e. ‘the ability to survive the dry state for prolonged periods of time’), are acquired at seed filling and the later maturation phase, respectively (Verdier *et al.*, 2013). ABI3 has been shown to be involved in both desiccation tolerance and seed longevity. The severe *abi3* mutant alleles such as *abi3-4* and *abi3-6*, which have a short form of ABI3 due to a mutation-induced premature stop codon, are intolerant to seed desiccation. Two mutant alleles, *abi3-1* and *abi3-7*, which contain one or two amino acid substitutions in the B2 and B3 domain, are tolerant to seed desiccation but show reduction in seed longevity (Ooms *et al.*, 1993; Nambara *et al.*, 1994; Bies-Etheve *et al.*, 1999; Tesnier *et al.*, 2002). Other weak *abi3* alleles were not reported to have a reduction in seed longevity. The different effects may be related to the differences in downstream target genes affected.

A systematic analysis of the *Medicago* seed development process by transcriptomic and metabolomic profiling (Verdier *et al.*, 2013), as well as studies in *Arabidopsis*, revealed that LEA proteins are more closely related to acquisition of desiccation tolerance, whereas sHSPs function in desiccation tolerance and longevity (Wehmeyer and Vierling, 2000; Prieto-Dapena *et al.*, 2006). In *Arabidopsis*, ABI3 directly activates the expression of the transcription factor HSFA9, and HSFA9 activates the expression of sHSPs in seeds (Kotak *et al.*, 2007). Ectopic expression of *HaHSFA9* in *Arabidopsis* leads to the activation of sHSP expression and results in increased seed longevity as well as enhanced desiccation tolerance in seedlings (Prieto-Dapena *et al.*, 2006). Desiccation tolerance and longevity pathways are also connected and share common components. Several lines of evidence suggest that some (but not all) LEA proteins are implicated in seed longevity. In *Arabidopsis*, a reduction in the levels of three seed-expressed dehydrins results in decreased longevity (Hundertmark *et al.*, 2011). In *Medicago*, the four most abundant seed LEA proteins are correlated with longevity (Chatelain *et al.*, 2012). These LEA genes are highly abundant in seeds and also regulated by the ABI3 transcription factor. ABI3 therefore functions as a master regulator that regulates the expression of genes in seeds, including *LEA* and *sHSP* genes, and controls both the desiccation tolerance and longevity pathways (Prieto-Dapena *et al.*, 2006; Hundertmark *et al.*, 2011).

Here, *TIP3;1* and *TIP3;2* were identified as members of ABI3 target genes, and TIP3s were added as new components in the seed longevity regulatory network. *TIP3* genes are direct targets of ABI3, as demonstrated by ChIP-chip analysis (Monke *et al.*, 2012). Systematic analyses was carried out to demonstrate that *TIP3;1* and *TIP3;2* are target genes of the ABI3 transcription factor, providing evidence that the B3 domain of ABI3 can bind directly to the RY motifs in the *TIP3* promoters; ABI3 is critical for the *TIP3* promoter activity in response to ABA and *TIP3* gene expression in mature seeds (Figs 5, 6). ABI3 therefore plays a critical role in seed longevity through the expressional regulation of *TIP3*, *sHSP*, and *LEA* genes.

### TIP3 transport function and seed longevity

Seed longevity is an important genetic trait for preservation of seed viability and seed quality during storage. Orthodox seeds keep their capacity to germinate before and after storage, but gradually lose their viability during storage, which is influenced by genetic factors and environmental factors. Seed storage temperature and seed moisture content are the two most important factors that control seed deterioration and viability loss during storage (Roberts and Ellis, 1989; Bradford *et al.*, 1993; McDonald, 1999). The underlying mechanism of TIP3s in maintaining seed longevity is not clear. TIP3;2 has dual activities on water and H_2_O_2_ permeability (Fig. 7; Supplementary Fig. S9 at *JXB* online). Some PIPs and TIPs possess the function to facilitate the permeation of H_2_O_2_ across membranes, as demonstrated by growth and survival assays with yeast cells expressing AQPs and by H_2_O_2_-detecting fluorescence assays (reviewed by Bienert and Chaumont, 2014). These identified AQPs are mostly expressed in vegetative tissues, but the biological significance of H_2_O_2_ permeation was not addressed. Here it was found that seed-specific TIP3;2 but not TIP3;1 also mediated diffusion of H_2_O_2_ across the membrane in the yeast system. ROS are detrimental to seed longevity due to their deteriorative effects on lipids, nucleic acids, and proteins. Vacuoles potentially have a function in ROS detoxification, but the direct evidence for this is still lacking (Mittler *et al.*, 2004; Smirnoff, 2005). TIP3;2 may be involved in H_2_O_2_ permeation and detoxification in seeds. However, the *tip3;1* or *tip3;2* single mutant does not show a significant decrease in seed longevity and only the *tip3;1*/*tip3;2* double mutant is very sensitive to artificial ageing and accumulates a higher amount of H_2_O_2_ in the CDT. This result suggests that TIP3;1 and TIP3;2 are functionally redundant in maintenance of seed longevity, and the decrease in seed longevity of the double mutant is not only caused by loss of H_2_O_2_ permeability of TIP3;2. The activities of TIP3s in water permeation are important for seed longevity, since the remaining 30% of *TIP3;1* expression in the *tip3;1*/*tip3;2* double mutant resulted in higher seed longevity than *TIP3;1*-RNAi/*tip3;2* (Figs 2D, 3C, E). Consistently, a low concentration of HgCl_2_, which is an inhibitor of AQP, also affect the basal thermotolerance of seeds (Supplementary Fig. S4A).

Water is an essential element during seed desiccation and seed germination, and plays a critical role in the regulation of various seed metabolic processes. Seed moisture content is an important factor for seed deterioration, and the appropriate moisture content can increase seed longevity (Roberts and Ellis, 1989; McDonald, 1999). Lipid auto-oxidation generates various ROS and causes seed deterioration at a moisture content <6%. Above a 14% moisture content, lipid oxidation may again be stimulated by the activity of hydrolytic oxidative enzymes (Labuza *et al.*, 1972; Roberts and Ellis, 1989; McDonald, 1999; Shaban, 2013). Moreover, under a high moisture content, antioxidant enzymes (such as catalase, superoxide dismutase, and glutathione reductase) gradually lose activity and ROS will be accumulated (Bailly *et al.*, 1996; Bailly, 2004). This suggests that changes in water relations from seed development to seed storage and stable water relations in mature seeds may be important for seed longevity. ROS generation may be caused by over-high and over-low seed moisture content. However, the seed moisture content of *tip3* double mutants is not significantly changed compared with that of WT mature seeds. Along with the changes in environmental humidity, TIP3s may mediate cell–cell and intracellular water transport and help embryo cells maintain stable water relations in prolonged storage or stressed conditions. Higher H_2_O_2_ accumulation in *tip3* double mutants might be caused by impaired water transport regulation during seed desiccation and seed storage as a result of loss of water permeability of TIP3s.

In the present study, evidence was provided that seed-specific TIP3;1 and TIP3;2 play a role in maintaining seed longevity during seed ageing. TIP3;2 but not TIP3;1 functions in H_2_O_2_ permeation. ABI3 plays the critical role in seed longevity through the expressional regulation of seed-specific gene expression. TIP3s are new members of ABI3 target genes during seed maturation, which work together with sHSPs and LEAs to control seed longevity.

## Supplementary data

Supplementary data are available at *JXB* online.

Figure S1. Immunoblot analysis of membrane proteins from yeast cells transformed with *AtTIP1;1*, *AtTIP2;1*, *AtTIP3;1*, *AtTIP3;2*, *AtTIP4;1*, *AtTIP5;1*, or empty vector pYX212.

Figure S2. Detection of the GFP fluorescence in *Pro*_*TIP3;1*_:*GFP* transgenic seeds.

Figure S3. Growth of *tip3;1*, *tip3;2*, and *tip3;1/tip3;2* mutants compared with Col.

Figure S4. Basal thermotolerance assays of Col, *tip3;1*, *tip3;2*, and *tip3;1/tip3;2* seeds.

Figure S5. qRT-PCR analysis of *ABI3* transcripts in *abi3-6* and *fus3-3* seeds.

Figure S6. Expression analysis of some seed-expressed genes in seeds of different *abi3* alleles.

Figure S7. The *TIP3;1* promoter is inactive in developing seeds and embryos of *abi3-6*.

Figure S8. ABI3 binds to *TIP3s* promoters containing RY motifs.

Figure S9. Yeast protoplast swelling assays.

Table S1. List of primers used in this study.

Supplementary Data

## Abbreviations:

ABA: abscisic acid
ABI3: ABSCISIC ACID INSENSITIVE 3
AQP: aquaporin
BTA: basal thermotolerance assay
CDT: controlled deterioration test
DAB: 3,3′-diaminobenzidine
DPA: days post-anthesis
LEA: late embryo abundant
PIP: plasma membrane intrinsic protein
PSV: protein storage vacuole
ROS: reactive oxygen species
sHSP: small heat shock protein
TIP: tonoplast intrinsic protein.

## References

[CIT0001] Bailly C 2004 Active oxygen species and antioxidants in seed biology. Seed Science Research 14, 93–107.

[CIT0002] Bailly C Benamar A Corbineau F Come D 1996 Changes in malondialdehyde content and in superoxide dismutase, catalase and glutathione reductase activities in sunflower seeds as related to deterioration during accelerated aging. Physiologia Plantarum 97, 104–110.

[CIT0003] Bienert GP Chaumont F 2014 Aquaporin-facilitated transmembrane diffusion of hydrogen peroxide. Biochimica et Biophysica Acta 1840, 1596–1604. 24060746 10.1016/j.bbagen.2013.09.017

[CIT0004] Bienert GP Heinen RB Berny MC Chaumont F 2014 Maize plasma membrane aquaporin ZmPIP2;5, but not ZmPIP1;2, facilitates transmembrane diffusion of hydrogen peroxide. Biochimica et Biophysica Acta 1838, 216–222. 23994602 10.1016/j.bbamem.2013.08.011

[CIT0005] Bienert GP Moller AL Kristiansen KA Schulz A Moller IM Schjoerring JK Jahn TP 2007 Specific aquaporins facilitate the diffusion of hydrogen peroxide across membranes. Journal of Biological Chemistry 282, 1183–1192. 17105724 10.1074/jbc.M603761200

[CIT0006] Bies-Etheve N da Silva Conceicao A Giraudat J Koornneef M Leon-Kloosterziel K Valon C Delseny M 1999 Importance of the B2 domain of the Arabidopsis ABI3 protein for Em and 2S albumin gene regulation. Plant Molecular Biology 40, 1045–1054. 10527428 10.1023/a:1006252512202

[CIT0007] Bradford KJ Tarquis AM Duran JM 1993 A population-based threshold-model describing the relationship between germination rates and seed deterioration. Journal of Experimental Botany 44, 1225–1234.

[CIT0008] Brand LH Kirchler T Hummel S Chaban C Wanke D 2010 DPI-ELISA: a fast and versatile method to specify the binding of plant transcription factors to DNA *in vitro* . Plant Methods 6, 25. 21108821 10.1186/1746-4811-6-25 PMC3003642

[CIT0009] Chatelain E Hundertmark M Leprince O Le Gall S Satour P Deligny-Penninck S Rogniaux H Buitink J 2012 Temporal profiling of the heat-stable proteome during late maturation of Medicago truncatula seeds identifies a restricted subset of late embryogenesis abundant proteins associated with longevity. Plant, Cell and Environment 35, 1440–1455. 10.1111/j.1365-3040.2012.02501.x 22380487

[CIT0010] Chaumont F Barrieu F Wojcik E Chrispeels MJ Jung R 2001 Aquaporins constitute a large and highly divergent protein family in maize. Plant Physiology 125, 1206–1215. 11244102 10.1104/pp.125.3.1206 PMC65601

[CIT0011] Chen H Chu P Zhou Y Li Y Liu J Ding Y Tsang EW Jiang L Wu K Huang S 2012 Overexpression of AtOGG1, a DNA glycosylase/AP lyase, enhances seed longevity and abiotic stress tolerance in Arabidopsis. Journal of Experimental Botany 63, 4107–4121. 22473985 10.1093/jxb/ers093

[CIT0012] Daniels MJ Chaumont F Mirkov TE Chrispeels MJ 1996 Characterization of a new vacuolar membrane aquaporin sensitive to mercury at a unique site. The Plant Cell 8, 587–599. 8624437 10.1105/tpc.8.4.587 PMC161122

[CIT0013] Delmas F Sankaranarayanan S Deb S Widdup E Bournonville C Bollier N Northey JG McCourt P Samuel MA 2013 ABI3 controls embryo degreening through Mendel’s I locus. Proceedings of the National Academy of Sciences, USA 110, E3888–E3894. 10.1073/pnas.1308114110 PMC3791760 24043799

[CIT0014] Dynowski M Schaaf G Loque D Moran O Ludewig U 2008 Plant plasma membrane water channels conduct the signalling molecule H_2_O_2_ . Biochemical Journal 414, 53–61. 18462192 10.1042/BJ20080287

[CIT0015] Gattolin S Sorieul M Frigerio L 2011 Mapping of tonoplast intrinsic proteins in maturing and germinating Arabidopsis seeds reveals dual localization of embryonic TIPs to the tonoplast and plasma membrane. Molecular Plant 4, 180–189. 20833734 10.1093/mp/ssq051

[CIT0016] Giraudat J Hauge BM Valon C Smalle J Parcy F Goodman HM 1992 Isolation of the Arabidopsis ABI3 gene by positional cloning. The Plant Cell 4, 1251–1261. 1359917 10.1105/tpc.4.10.1251 PMC160212

[CIT0017] Hellens RP Allan AC Friel EN Bolitho K Grafton K Templeton MD Karunairetnam S Gleave AP Laing WA 2005 Transient expression vectors for functional genomics, quantification of promoter activity and RNA silencing in plants. Plant Methods 1, 13. 16359558 10.1186/1746-4811-1-13 PMC1334188

[CIT0018] Hofte H Hubbard L Reizer J Ludevid D Herman EM Chrispeels MJ 1992 Vegetative and seed-specific forms of tonoplast intrinsic protein in the vacuolar membrane of Arabidopsis thaliana. Plant Physiology 99, 561–570. 16668923 10.1104/pp.99.2.561 PMC1080500

[CIT0019] Hooijmaijers C Rhee JY Kwak KJ Chung GC Horie T Katsuhara M Kang H 2012 Hydrogen peroxide permeability of plasma membrane aquaporins of Arabidopsis thaliana. Journal of Plant Research 125, 147–153. 21390558 10.1007/s10265-011-0413-2

[CIT0020] Hundertmark M Buitink J Leprince O Hincha DK 2011 The reduction of seed-specific dehydrins reduces seed longevity in Arabidopsis thaliana. Seed Science Research 21, 165–173.

[CIT0021] Jauh GY Fischer AM Grimes HD Ryan CA Rogers JC 1998 delta-Tonoplast intrinsic protein defines unique plant vacuole functions. Proceedings of the National Academy of Sciences, USA 95, 12995–12999. 10.1073/pnas.95.22.12995 PMC23684 9789029

[CIT0022] Johnson KD Herman EM Chrispeels MJ 1989 An abundant, highly conserved tonoplast protein in seeds. Plant Physiology 91, 1006–1013. 16667102 10.1104/pp.91.3.1006 PMC1062109

[CIT0023] Koornneef M Reuling G Karssen CM 1984 The isolation and characterization of abscisic-acid insensitive mutants of Arabidopsis thaliana. Physiologia Plantarum 61, 377–383.

[CIT0024] Kotak S Vierling E Baumlein H von Koskull-Doring P 2007 A novel transcriptional cascade regulating expression of heat stress proteins during seed development of Arabidopsis. The Plant Cell 19, 182–195. 17220197 10.1105/tpc.106.048165 PMC1820961

[CIT0025] Labuza TP Hawkes J Gallaghe D Hurtado F Mcnally L 1972 Stability of intermediate moisture foods. 1. Lipid oxidation. Journal of Food Science 37, 154–159.

[CIT0026] Lee SE Yim HK Lim MN Yoon IS Kim JH Hwang YS 2014 Abscisic acid prevents the coalescence of protein storage vacuoles by upregulating expression of a tonoplast intrinsic protein gene in barley aleurone. Journal of Experimental Botany 66, 1191–1203. 25477530 10.1093/jxb/eru467 PMC4438444

[CIT0027] Li G Santoni V Maurel C 2013 Plant aquaporins: roles in plant physiology. Biochimica et Biophysica Acta 1840, 1574–1582. 24246957 10.1016/j.bbagen.2013.11.004

[CIT0028] Liu LH Ludewig U Gassert B Frommer WB von Wiren N 2003 Urea transport by nitrogen-regulated tonoplast intrinsic proteins in Arabidopsis. Plant Physiology 133, 1220–1228. 14576283 10.1104/pp.103.027409 PMC281617

[CIT0029] Loque D Ludewig U Yuan L von Wiren N 2005 Tonoplast intrinsic proteins AtTIP2;1 and AtTIP2;3 facilitate NH3 transport into the vacuole. Plant Physiology 137, 671–680. 15665250 10.1104/pp.104.051268 PMC1065367

[CIT0030] Ludevid D Hofte H Himelblau E Chrispeels MJ 1992 The expression pattern of the tonoplast intrinsic protein gamma-TIP in Arabidopsis thaliana is correlated with cell enlargement. Plant Physiology 100, 1633–1639. 16653178 10.1104/pp.100.4.1633 PMC1075845

[CIT0700] Mao J, Zhang YC, Sang Y, Li QH, Yang HQ. 2005. A role for Arabidopsis cryptochromes and COP1 in the regulation of stomatal opening. Proceedings of the National Academy of Sciences, USA 102, 12270–12275. 10.1073/pnas.0501011102 PMC1189306 16093319

[CIT0031] Martre P North GB Nobel PS 2001 Hydraulic conductance and mercury-sensitive water transport for roots of Opuntia acanthocarpa in relation to soil drying and rewetting. Plant Physiology 126, 352–362. 11351098 10.1104/pp.126.1.352 PMC102309

[CIT0032] Maurel C Chrispeels MJ 2001 Aquaporins. A molecular entry into plant water relations. Plant Physiology 125, 135–138. 11154316 10.1104/pp.125.1.135 PMC1539345

[CIT0033] Maurel C Kado RT Guern J Chrispeels MJ 1995 Phosphorylation regulates the water channel activity of the seed-specific aquaporin alpha-TIP. EMBO Journal 14, 3028–3035. 7542585 10.1002/j.1460-2075.1995.tb07305.x PMC394363

[CIT0034] McCarty DR Hattori T Carson CB Vasil V Lazar M Vasil IK 1991 The Viviparous-1 developmental gene of maize encodes a novel transcriptional activator. Cell 66, 895–905. 1889090 10.1016/0092-8674(91)90436-3

[CIT0035] McDonald MB 1999 Seed deterioration: physiology, repair and assessment. Seed Science and Technology 27, 177–237.

[CIT0036] Mittler R Vanderauwera S Gollery M Van Breusegem F 2004 Reactive oxygen gene network of plants. Trends in Plant Science 9, 490–498. 15465684 10.1016/j.tplants.2004.08.009

[CIT0037] Monke G Seifert M Keilwagen J 2012 Toward the identification and regulation of the Arabidopsis thaliana ABI3 regulon. Nucleic Acids Research 40, 8240–8254. 22730287 10.1093/nar/gks594 PMC3458547

[CIT0038] Nambara E Keith K McCourt P Naito S 1994 Isolation of an internal deletion mutant of the Arabidopsis thaliana ABI3 gene. Plant and Cell Physiology 35, 509–513. 8055176

[CIT0039] Oge L Bourdais G Bove J Collet B Godin B Granier F Boutin JP Job D Jullien M Grappin P 2008 Protein repairl-isoaspartyl methyltransferase 1 is involved in both seed longevity and germination vigor in Arabidopsis. The Plant Cell 20, 3022–3037. 19011119 10.1105/tpc.108.058479 PMC2613667

[CIT0040] Ooms J Leon-Kloosterziel KM Bartels D Koornneef M Karssen CM 1993 Acquisition of desiccation tolerance and longevity in seeds of Arabidopsis thaliana (a comparative study using abscisic acid-insensitive abi3 mutants). Plant Physiology 102, 1185–1191. 12231895 10.1104/pp.102.4.1185 PMC158904

[CIT0041] Parcy F Valon C Raynal M Gaubier-Comella P Delseny M Giraudat J 1994 Regulation of gene expression programs during Arabidopsis seed development: roles of the ABI3 locus and of endogenous abscisic acid. The Plant Cell 6, 1567–1582. 7827492 10.1105/tpc.6.11.1567 PMC160544

[CIT0042] Park J Lee N Kim W Lim S Choi G 2011 ABI3 and PIL5 collaboratively activate the expression of SOMNUS by directly binding to its promoter in imbibed Arabidopsis seeds. The Plant Cell 23, 1404–1415. 21467583 10.1105/tpc.110.080721 PMC3101561

[CIT0043] Prieto-Dapena P Castano R Almoguera C Jordano J 2006 Improved resistance to controlled deterioration in transgenic seeds. Plant Physiology 142, 1102–1112. 16998084 10.1104/pp.106.087817 PMC1630740

[CIT0044] Reuscher S Akiyama M Mori C Aoki K Shibata D Shiratake K 2013 Genome-wide identification and expression analysis of aquaporins in tomato. PLoS One 8, e79052. 24260152 10.1371/journal.pone.0079052 PMC3834038

[CIT0045] Roberts EH Ellis RH 1989 Water and seed survival. Annals of Botany 63, 39–52.

[CIT0046] Rohde A De Rycke R Beeckman T Engler G Van Montagu M Boerjan W 2000 ABI3 affects plastid differentiation in dark-grown Arabidopsis seedlings. The Plant Cell 12, 35–52. 10634906 10.1105/tpc.12.1.35 PMC140213

[CIT0047] Roschzttardtz H Fuentes I Vasquez M Corvalan C Leon G Gomez I Araya A Holuigue L Vicente-Carbajosa J Jordana X 2009 A nuclear gene encoding the iron–sulfur subunit of mitochondrial complex II is regulated by B3 domain transcription factors during seed development in Arabidopsis. Plant Physiology 150, 84–95. 19261733 10.1104/pp.109.136531 PMC2675723

[CIT0048] Sakurai J Ishikawa F Yamaguchi T Uemura M Maeshima M 2005 Identification of 33 rice aquaporin genes and analysis of their expression and function. Plant and Cell Physiology 46, 1568–1577. 16033806 10.1093/pcp/pci172

[CIT0049] Sattler SE Gilliland LU Magallanes-Lundback M Pollard M DellaPenna D 2004 Vitamin E is essential for seed longevity and for preventing lipid peroxidation during germination. The Plant Cell 16, 1419–1432. 15155886 10.1105/tpc.021360 PMC490036

[CIT0050] Savage DF Stroud RM 2007 Structural basis of aquaporin inhibition by mercury. Journal of Molecular Biology 368, 607–617. 17376483 10.1016/j.jmb.2007.02.070 PMC3535476

[CIT0051] Shaban M 2013 Review on physiological aspects of seed deterioration. International Journal of Agriculture and Crop Sciences 6, 627–631.

[CIT0052] Smirnoff N 2005 Antioxidants and reactive oxygen species in plants. Oxford: Wiley-Blackwell Publishing.

[CIT0053] Suzuki M Kao CY McCarty DR 1997 The conserved B3 domain of VIVIPAROUS1 has a cooperative DNA binding activity. The Plant Cell 9, 799–807. 9165754 10.1105/tpc.9.5.799 PMC156957

[CIT0054] Suzuki M McCarty DR 2008 Functional symmetry of the B3 network controlling seed development. Current Opinion in Plant Biology 11, 548–553. 18691932 10.1016/j.pbi.2008.06.015

[CIT0055] Tesnier K Strookman-Donkers HM Van Pijlen JG Van der Geest AHM Bino RJ Groot SPC 2002 A controlled deterioration test for Arabidopsis thaliana reveals genetic variation in seed quality. Seed Science and Technology 30, 149–165.

[CIT0056] Vander Willigen C Postaire O Tournaire-Roux C Boursiac Y Maurel C 2006 Expression and inhibition of aquaporins in germinating Arabidopsis seeds. Plant and Cell Physiology 47, 1241–1250. 16926168 10.1093/pcp/pcj094

[CIT0057] Vandesompele J De Preter K Pattyn F Poppe B Van Roy N De Paepe A Speleman F 2002 Accurate normalization of real-time quantitative RT-PCR data by geometric averaging of multiple internal control genes. Genome Biology 3, RESEARCH0034. 10.1186/gb-2002-3-7-research0034 PMC126239 12184808

[CIT0058] Verdier J Lalanne D Pelletier S 2013 A regulatory network-based approach dissects late maturation processes related to the acquisition of desiccation tolerance and longevity of Medicago truncatula seeds. Plant Physiology 163, 757–774. 23929721 10.1104/pp.113.222380 PMC3793056

[CIT0059] Verma P Kaur H Petla BP Rao V Saxena SC Majee M 2013 PROTEIN L-ISOASPARTYL METHYLTRANSFERASE2 is differentially expressed in chickpea and enhances seed vigor and longevity by reducing abnormal isoaspartyl accumulation predominantly in seed nuclear proteins. Plant Physiology 161, 1141–1157. 23284083 10.1104/pp.112.206243 PMC3585586

[CIT0060] Wang YY Hecker AG Hauser BA 2014 The APX4 locus regulates seed vigor and seedling growth in Arabidopsis thaliana. Planta 239, 909–919. 24407512 10.1007/s00425-014-2025-2

[CIT0061] Waterworth WM Masnavi G Bhardwaj RM Jiang Q Bray CM West CE 2010 A plant DNA ligase is an important determinant of seed longevity. The Plant Journal 63, 848–860. 20584150 10.1111/j.1365-313X.2010.04285.x

[CIT0062] Wehmeyer N Hernandez LD Finkelstein RR Vierling E 1996 Synthesis of small heat-shock proteins is part of the developmental program of late seed maturation. Plant Physiology 112, 747–757. 8883386 10.1104/pp.112.2.747 PMC157999

[CIT0063] Wehmeyer N Vierling E 2000 The expression of small heat shock proteins in seeds responds to discrete developmental signals and suggests a general protective role in desiccation tolerance. Plant Physiology 122, 1099–1108. 10759505 10.1104/pp.122.4.1099 PMC58944

[CIT0064] Wharton MJ 1955 The use of tetrazolium test for determining the viability of seeds of the genus Brassica. Proceedings of the National Academy of Sciences, USA 20, 81–88.

[CIT0065] Wu FH Shen SC Lee LY Lee SH Chan MT Lin CS 2009 Tape–Arabidopsis Sandwich—a simpler Arabidopsis protoplast isolation method. Plant Methods 5, 16. 19930690 10.1186/1746-4811-5-16 PMC2794253

[CIT0066] Wudick MM Luu DT Tournaire-Roux C Sakamoto W Maurel C 2014 Vegetative and sperm cell-specific aquaporins of Arabidopsis highlight the vacuolar equipment of pollen and contribute to plant reproduction. Plant Physiology 164, 1697–1706. 24492334 10.1104/pp.113.228700 PMC3982734

[CIT0067] Yoo SD Cho YH Sheen J 2007 Arabidopsis mesophyll protoplasts: a versatile cell system for transient gene expression analysis. Nature Protocols 2, 1565–1572. 17585298 10.1038/nprot.2007.199

[CIT0068] Zhang DY Ali Z Wang CB 2013 Genome-wide sequence characterization and expression analysis of major intrinsic proteins in soybean (Glycine max L.). PLoS One 8, e56312. 23437113 10.1371/journal.pone.0056312 PMC3577755

