# Supplementary material for: Arabidopsis seed-specific vacuolar aquaporins are involved in maintaining seed longevity under the control of ABSCISIC ACID INSENSITIVE 3
Source: J Exp Bot. 2015 May 26;66(15):4781–94. doi: 10.1093/jxb/erv244 (PMC4507774; doi:10.1093/jxb/erv244)
Supplement: Supplementary Data [file supp_66_15_4781__index.html]

 Arabidopsis seed-specific vacuolar aquaporins are involved in maintaining seed longevity under the control of ABSCISIC ACID INSENSITIVE 3 — Arabidopsis seed-specific vacuolar aquaporins are involved in maintaining seed longevity under the control of ABSCISIC ACID INSENSITIVE 3 — Supplementary Data 

# *Arabidopsis* seed-specific vacuolar aquaporins are involved in maintaining seed longevity under the control of *ABSCISIC ACID INSENSITIVE 3*

## Supplementary Data

Data files

- Supplementary Data - Supplementary Data
