# Supplementary material for: Arabidopsis seed-specific vacuolar aquaporins are involved in maintaining seed longevity under the control of ABSCISIC ACID INSENSITIVE 3
Source: J Exp Bot. 2015 May 26;66(15):4781–94. doi: 10.1093/jxb/erv244 (PMC4507774; doi:10.1093/jxb/erv244)
Supplement: Supplementary Data [file supp_erv244_jexbot144550_file001.pdf]

Supplementary Data

Supplementary table 1 List of primers used in this study.

Name	Forward primer	Reverse primer	Purposes
TIP3;1	CGGAAGCTTATGGCAACATCAGCTCGTAGAG	CGGCTGCAGGTAATCTTCAGGGGCCAAGG	gene clone
TIP3;2	CGGAAGCTTATGGCTACATCTGCTAGAAGAG	GGATCCGTAATCTTCCGGAGCCAATGGTTG	gene clone
ABI3	GGATCCATGAAAAGCTTGCATGTGG	TCTAGACATTTAACAGTTTGAGAAG	gene clone
FUS3	AGAGAACTGTTTTCTTCCAC	AAGAATACCATGAGCCCATC	gene clone
PIP2;5	cggGAATTCATGACGAAGGAAGTGGTTGG	cggGGATCCTTAAACGTGAGGCTGGC	gene clone
TIP1;1	cggGAATTCATGCCGATCAGAAACATC	cggAAGCTTTCAGTAGTCTGTGGTTGGGAGCTG	gene clone
TIP2;1	cggGAATTCATGGCTGGAGTTGCCTTTG	cggAAGCTTTTAGAAATCAGCAGAAGC	gene clone
TIP4;1	cggAAGCTTATGAAGAAGATCGAGTTAGGG	cggCCCGGGTTAATTCAACAATGGTTGCTC	gene clone
TIP5;1	cggGAATTCATGAGAAGAATGATTCCAACATCG	cggGGATCCTTACACACCAATGGCATCACC	gene clone
$Pro_{TIP3;1}$	AAGCTTTATACTCCTTCTGGCATTGC	CCCGGGAGTGTGAAGAAGAAAGAGAG	promoter
$Pro_{TIP3;2}$	AAGCTTCAATCATCTGGACACGTTTC	CCCGGGAAGTGAGAGGATCAAACC	promoter
mRY1	CTCCGCATGCTAAGTGACACGCTGCTGTTCAAGCC	GTGTCACTTAGCATGCGGAGGTCGATATGCGGCC	promoter
mRY2	TATCTACTTGGCACATGACACTGCTTAGTCAACAC	GTGTCATGTGCCAAGTAGATAAACAAGCATATGAACCT	promoter
mRY3	TGGTTAAGTAATTGTGACACATTTAAAGCTACGTG	GTGTCACAATTACTTAACCATAACTTAGATG	promoter
TIP31-qRT	CCCACCGAACCACCTACC	GAACAACGAACAAAAGCA	qRT-PCR
TIP32-qRT	ACCACAGTACCCACCAAC	ACATAGGAAATGGCAGGA	qRT-PCR
HSP17.6	AAACGCAAAGAGTAGCAATC	AAACATCCAGCGAGAACG	qRT-PCR
HSP17.7	ATAAGAACCGTTACATCGTCG	TATCCGCTCGCTTCATCG	qRT-PCR
HSFA9	ATGGCTGCAACAGAACAC	CCACCGTCAACAAGTAGG	qRT-PCR
PP2A-qRT	TATCGGATGACGATTCTTCGTGCAG	GCTTGGTCGACTATCGGAATGAGAG	qRT-PCR

ABI3-qRT	GTGGTCGCTTCACCAACTTCTC	CAGCTTTAATCATGACCCTC CA	qRT-PCR
GUS-qRT	GCCGATGCAGATATTCGTAATTATGCGGGCAAC	TTCAAATGGCGTATAGCCGCCCTGATGCTCCAT	qRT-PCR
At1g17210-qRT	CTGCTTCATATGAATCACGAG	TCAACACTATCTGCACGTTGT	qRT-PCR
At2g04660-qRT	TTCTGGAAGCAGTGGGTGAA	CTCCACTTCCATCTGTAAGC	qRT-PCR
At2g20000-qRT	GTATAGCTCCACCACCACTT	TCTTCTAGGTGCTTGAAGAGT	qRT-PCR
At4g04320-qRT	ACTCGGTATGTGGCTTAGTC	TTCTCAAGCAATGAAGCAGGA	qRT-PCR
Ef1a-qRT	TGAGCACGCTCTTCTTGCT	GTGGCATCCATCTTGTTACA	qRT-PCR
CYP5-qRT	CTTCAGAGCTTTGTGCACAGG	AAGCTGGGAATGATTCGATG	qRT-PCR
ACT7-qRT	GGTCGTACAACCGGTATTGT	GATAGCATGTGGAAGTGAGAA	qRT-PCR
ACT8-qRT	GGTCGTACAACCGGTATTGT	GAAGAGCATACCCCTCGTA	qRT-PCR
EM1-qRT	CGAGCTACTAGTGTCCGCTGCA	GTAAAACCAACCGGCAACCGCA	qRT-PCR
31pro	CGGGGTACCGGACAAGGATCAGCTC	CGGCCATGGTATGATCAAACACTTG	0800-LUC
32pro	CGGAAGCTTGATTGTGGTAAACCGAACTG	CGGCCATGGATATAACCCTAATTTAAGTGAGAGG	0800-LUC
ABI3	GGATCCATGAAAAGCTTGCATGTGG	GTCGACTGCCCTCTTTCTTATTTGG	62-SK
FUS3	GTCGACCATGATGGTTGATGAAAATGTG	GGTACCCTAGTAGAAGTCATCGAG	62-SK
ABI3-B3	CGGGAATTCCGGCAGGGATGGAAACCAG	CGGGCGGCCGCTTTAACAGTTTGAGAAGTTGG	Protein
ABI3-GST	GGATCCATGAAAAGCTTGCATGTGG	GTCGACTGCCCTCTTTCTTATTTGG	Protein
31RY2-bio	ATCTACTTGGCACACATGCATGCTTAGTCAACACA	TGTGTTGACTAAGCATGCATGTGTGCCAAGTAGAT	EMSA and DPI Elisa
31RY2-cold	ATCTACTTGGCACACATGCATGCTTAGTCAACACA	TGTGTTGACTAAGCATGCATGTGTGCCAAGTAGAT	EMSA
31RY2-mu	ATCTACTTGGCACAGCGCTAGCCTTAGTCAACACA	TGTGTTGACTAAGGCTAGCGCTGTGCCAAGTAGAT	EMSA
31RY2bio-mu	ATCTACTTGGCACAGCGCTAGCCTTAGTCAACACA	TGTGTTGACTAAGGCTAGCGCTGTGCCAAGTAGAT	DPI-Elisa
32RY-bio	CTTGATCCACTTGGCACACATGCATAGATATATAGTC	GCTGTGACTATATATCTATGCATGTGTGCCAAGTGGA	EMSA

	ACAGC	TCAAG	
32RY-cold	CTTGATCCACTTGGCACACATGCATAGATATATAGTCACAGC	GCTGTGACTATATATCTATGCATGTGTGCCAAGTGGATCAAG	EMSA
32RY-mu	CTTGATCCACTTGGCACAGCGTACTAGATATATAGTCACAGC	GCTGTGACTATATATCTAGTACGCTGTGCCAAGTGGATCAAG	EMSA
31RY	AATTCGGCACACATGCATGCTTAGTGGCACACATGCATGCTTAGTGGCACACATGCATGCTTAGTT	CTAGAACTAAGCATGCATGTGTGCCACTAAGCATGCATGTGTGCCACTAAGCATGCATGTGTGCCG	Y1H
32RY	AATTCCTTGGCACACATGCATAGATATATCTTGGCACACATGCATAGATATATCTTGGCACACATGCATAGATATATT	CTAGAATATATCTATGCATGTGTGCCAAGATATATCTATGCATGTGTGCCAAGATATATCTATGCATGTGTGCCAAGG	Y1H
ABI3	CGGGAATTCCGGCAGGGATGGAAACCAG	CGGGGATCCTCATTTAACAGTTTGAGAAG	Y1H
31RNAi	GGATCCCTGCAGTTTGGTCCAGCGTTGGTG	AAGCTTGAGCTCTCGAGCATTGCGAAATCG	RNAi
PDK-intron	GGATCCTTCGGTACCCCAGCTTGG	CTGCAGCCCAATTTCCCAACTG	RNAi
tip3;1	TTGATGGAGACTCCTCTGGTG	CGCTACTAAAATCAGCCCTCC	T-DNA mutants
tip3;2	AGGCTCTAGCCGGATTCATAG	CACGTGTTCAGCTTCAGACAC	

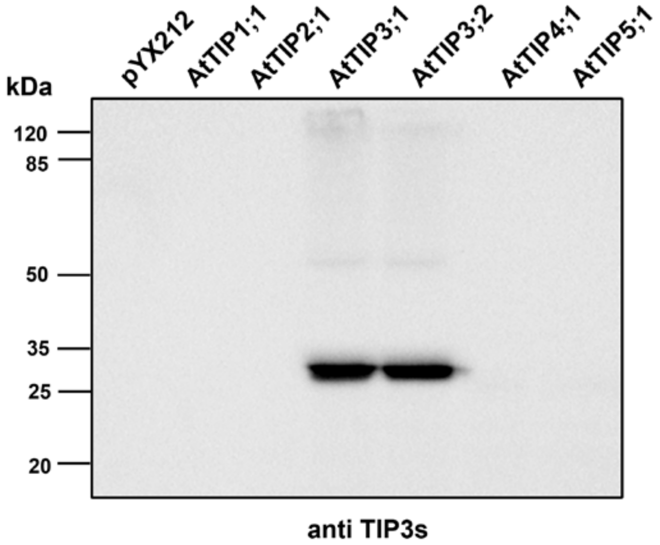

Supplementary Fig. S1 Immunoblot analysis of membrane proteins from yeast cells transformed with *AtTIP1;1*, *AtTIP2;1*, *AtTIP3;1*, *AtTIP3;2*, *AtTIP4;1*, *AtTIP5;1* or empty vector pYX212. Membrane proteins were separated by SDS-PAGE and blotted onto PVDF membrane. Anti-TIP3s antibodies were used in western blot analysis. Only TIP3;1 and TIP3;2 could be detected by the antibody.

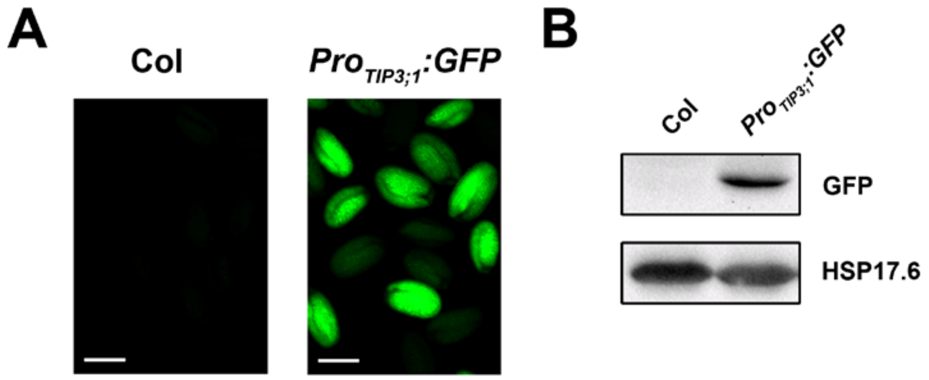

Supplementary Fig. S2 Detection of the GFP fluorescence in *Pro*$_{TIP3;1}$*:GFP* transgenic seeds.

(A) A 2-kb fragment of the *TIP3;1* promoter was fused to the *GFP* reporter gene and transformed into *Arabidopsis*. GFP fluorescent images were obtained under a fluorescence microscope. Mature seeds of WT (Col) were used as a negative control. Bar = 400 μm.

(B) Immunoblot analysis of GFP protein in *Pro*$_{TIP3;1}$*:GFP* transgenic seeds is shown on the right panel. Seed-expressed HSP17.6 protein was used as a loading control.

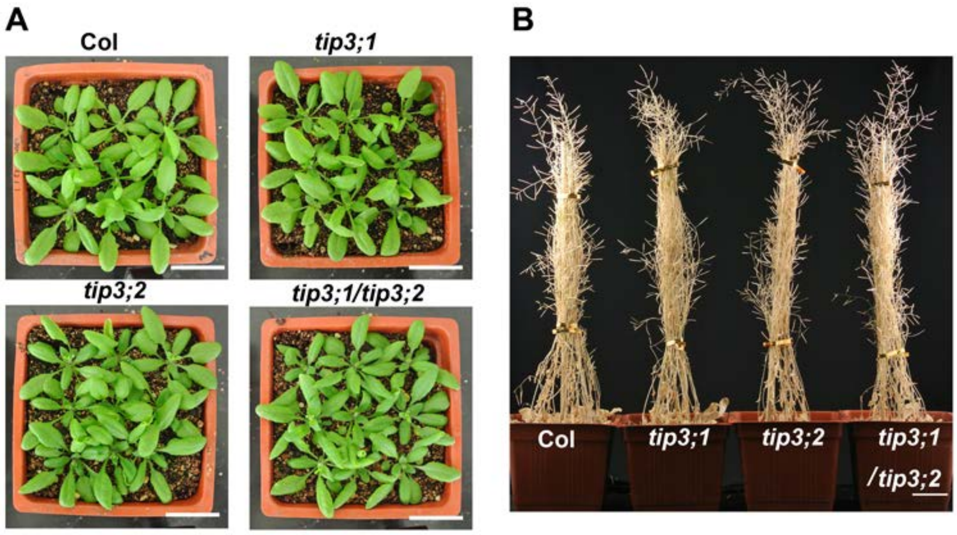

Supplementary Fig. S3 Growth of *tip3;1*, *tip3;2* and *tip3;1/tip3;2* mutants compared with Col. Col, *tip3;1*, *tip3;2* and *tip3;1/tip3;2*. Plants were photographed after one month **(A)** and two months of growth in soil **(B)**. Bars = 2 cm in (A) and 3 cm in (B).

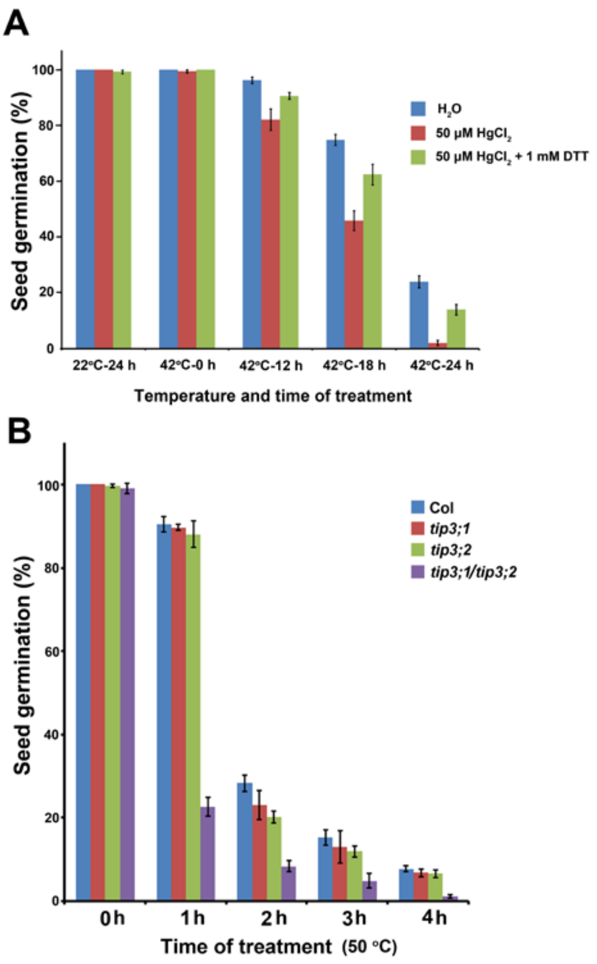

Supplementary Fig. S4 Basal thermotolerance assays of Col, *tip3;1*, *tip3;2* and *tip3;1 tip3;2* seeds.

(A) $HgCl_2$ treatment affects seed longevity. Imbibed seeds were incubated at 42 °C for different hours in the presence of 50 μM $HgCl_2$ or 1 mM DTT, washed five times with sterile water and then grown on MS medium at 22°C.

(B) Seeds used for testing were harvested at the same time, dried and stored under the same conditions for at least 2 weeks prior to the experiment. The imbibed seeds were incubated at 50°C for 1–4 h before germination at 22°C.

The germination percentages were counted 7 d after germination. Values are means ± SD of four technical replicates with 100 seeds per replicate.

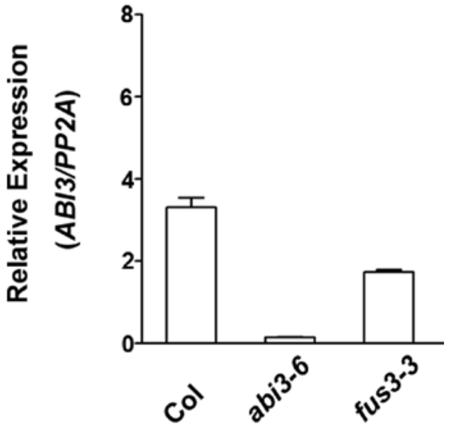

Supplementary Fig. S5 qRT-PCR analysis of *ABI3* transcripts in *abi3-6* and *fus3-3* seeds. Values are means ± SD, n = 3.

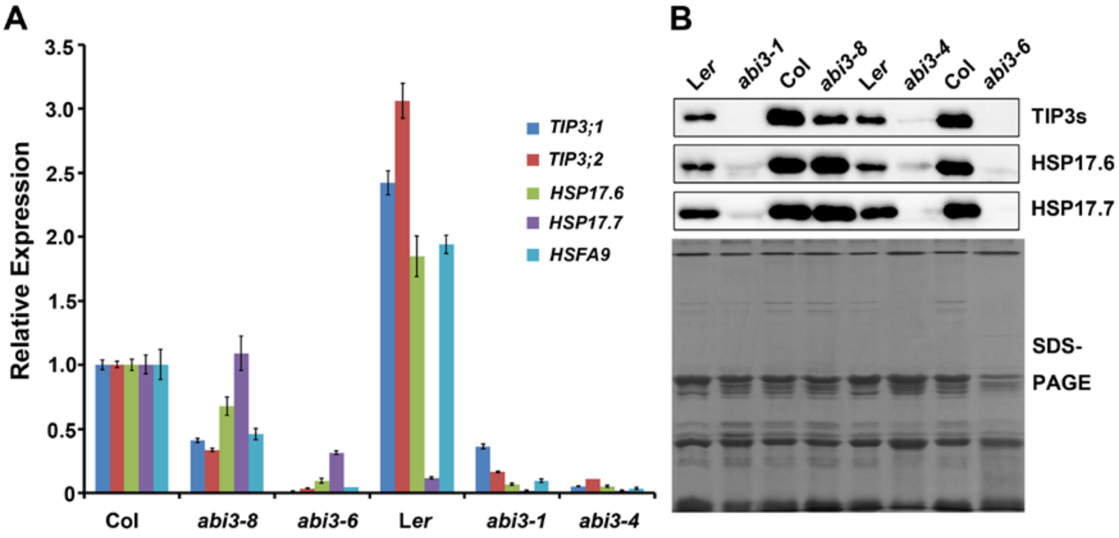

Supplementary Fig S6 Expression analysis of some seed-expressed genes in seeds of different *abi3* alleles .

(A) qRT-PCR analysis of *TIP3;1*, *TIP3;2*, *HSP17.6*, *HSP17.7* and *HSFA9* in the seeds of *abi3* mutants. The relative expression level of each gene was normalized to an endogenous control *PP2A* (At1g13320) and calculated using the $^{\Delta\Delta}$CT method.

(B) Immunoblot analysis of TIP3s, HSP17.6 and HSP17.7 proteins in the seeds of different *abi3* mutants. Similar amounts of proteins separated by SDS-PAGE were stained with Coomassie Brilliant Blue and used as a loading control.

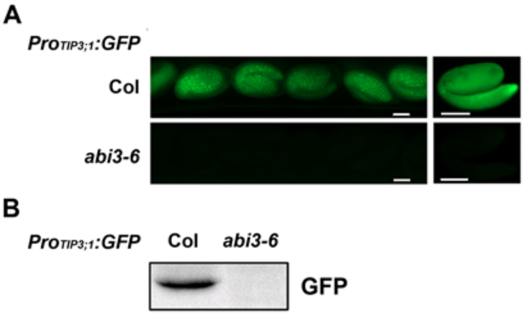

Supplementary Fig. S7 The *TIP3;1* promoter is inactive in developing seeds and embryos of *abi3-6*.

(A) The *TIP3;1* promoter is inactive in developing seeds and embryos of *abi3-6*. Comparison of GFP fluorescence in the developing seeds of $Pro_{TIP3;1}$:*GFP* transgenic plants with WT (Col) or *abi3-6* background. Left panel shows GFP fluorescence from the developing seeds at 18 DPA in a detached silique with the valve removed. Right panel shows GFP fluorescence from an embryo at 18 DPA isolated from a developing seed. Bar = 200 µm.

(B) Immunoblot analysis of GFP protein in mature seeds of $Pro_{TIP3;1}$:*GFP* transgenic plants with Col or *abi3-6* background.

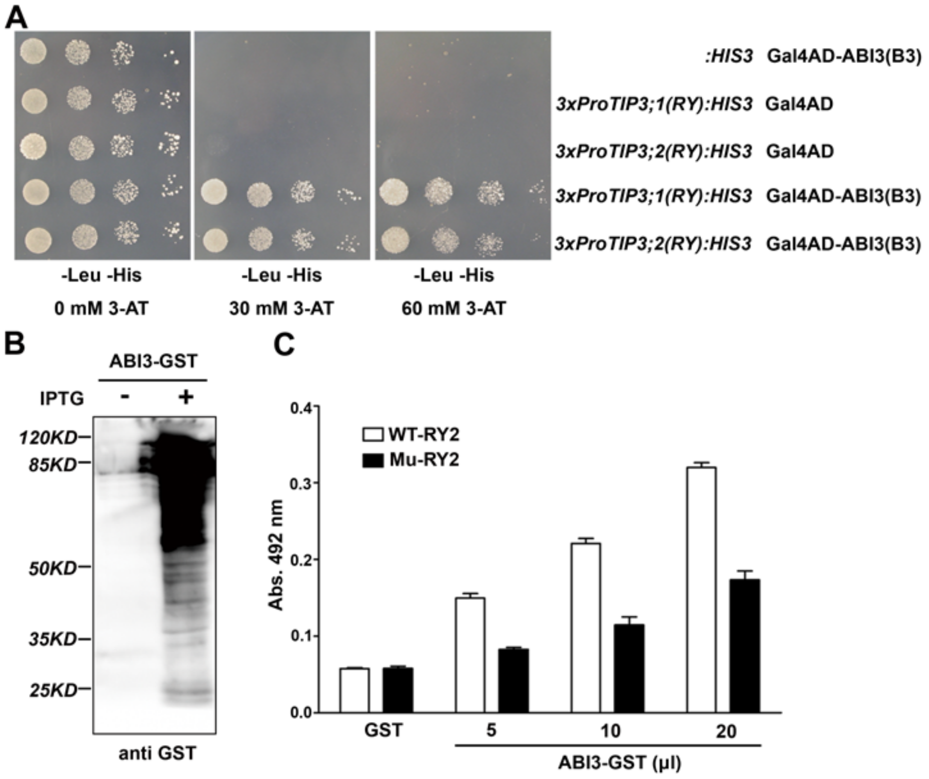

Supplementary Fig. S8 ABI3 binds to *TIP3s* promoters containing RY motifs.

(A) Yeast one-hybrid assay showing that the B3 domain of ABI3 binds to *TIP3* promoters. Three tandem copies of RY2 motifs from Pro:$_{TIP3;1}$ and RY motif from Pro:$_{TIP3;2}$ were fused upstream of the reporter gene *HIS3*. The interactions were detected on medium containing 30 mM or 60 mM 3-AT.

(B) Immunoblot analysis of recombinant ABI3-GST proteins expressed in *E. coli* before (-) and after (+) induction with 0.1 mM IPTG.

(C) DPI-ELISA analysis of the binding of full-length ABI3 protein to the WT and mutant RY2 motifs of the *TIP3;1* promoter. Different amounts of ABI3-GST recombinant proteins were used. The numbers indicate the volumes of *E. coli* extract used in the assay. Binding efficiency was measured by ELISA with an HRP conjugated anti-GST antibody. Control binding reactions were performed with extract from *E. coli* cells transformed with empty vector pGEX-4T-1.

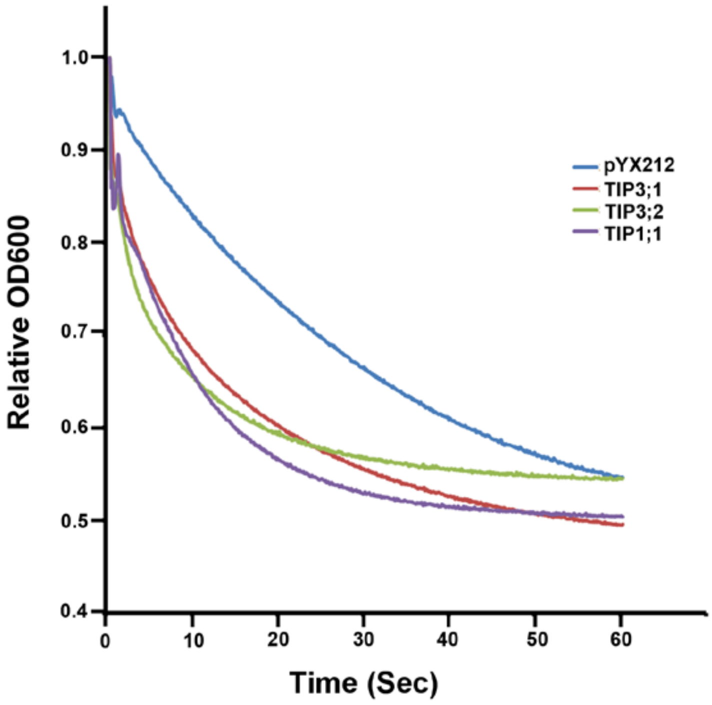

Supplementary Fig. S9 Yeast protoplasts swelling assays. Yeast protoplast expressing *TIP3;1* and *TIP3;2* burst much quicker than control protoplasts when exposed to a hypo-osmotic shock from 1.0 to 0.5 M sorbitol. The OD600 values were recorded per 0.1 sec.
